# Spatial Variation in Soil Fungal Communities across Paddy Fields in Subtropical China

**DOI:** 10.1128/mSystems.00704-19

**Published:** 2020-01-07

**Authors:** Pengfa Li, Weitao Li, Alex J. Dumbrell, Ming Liu, Guilong Li, Meng Wu, Chunyu Jiang, Zhongpei Li

**Affiliations:** a State Key Laboratory of Soil and Sustainable Agriculture, Institute of Soil Science, Chinese Academy of Sciences, Nanjing, China; b University of Chinese Academy of Sciences, Beijing, China; c School of Life Sciences, University of Essex, Colchester, Essex, United Kingdom; University of North Carolina at Chapel Hill

**Keywords:** DDRs, assemblages, fungi, paddy soils, soil profile, spatial variation

## Abstract

Fungi underpin almost all terrestrial ecosystem functions, yet our understanding of their community ecology lags far behind that of other organisms. Here, red paddy soils in subtropical China were collected across a soil depth profile, comprising 0-to-10-cm- (0-10cm-), 10-20cm-, and 20-40cm-deep layers. Using Illumina MiSeq amplicon sequencing of the internal transcribed spacer (ITS) region, distance-decay relationships (DDRs), and ecological models, fungal assemblages and their spatial patterns were investigated from each soil depth. We observed significant spatial variation in fungal communities and found that environmental heterogeneity decreased with soil depth, while spatial variation in fungal communities showed the opposite trend. DDRs occurred only in 0-10cm- and 10-20cm-deep soil layers, not in the 20-40cm layer. Our analyses revealed that the fungal community assembly in the 0-10cm layer was primarily governed by environmental filtering and a high dispersal rate, while in the deeper layer (20-40cm), it was primarily governed by dispersal limitation with minimal environmental filtering. Both environmental filtering and dispersal limitation controlled fungal community assembly in the 10-20cm layer, with dispersal limitation playing the major role. Results demonstrate the decreasing importance of environmental filtering and an increase in the importance of dispersal limitation in structuring fungal communities from shallower to deeper soils. Effectively, “everything is everywhere, but the environment selects,” although only in shallower soils that are easily accessible to dispersive fungal propagules. This work highlights that perceived drivers of fungal community assembly are dependent on sampling depth, suggesting that caution is required when interpreting diversity patterns from samples that integrate across depths.

**IMPORTANCE** In this work, Illumina MiSeq amplicon sequencing of the ITS region was used to investigate the spatial variation and assembly mechanisms of fungal communities from different soil layers across paddy fields in subtropical China, and the results demonstrate the decreasing importance of environmental filtering and an increase in the importance of dispersal limitation in structuring fungal communities from shallower to deeper soils. Therefore, the results of this study highlight that perceived drivers of fungal community assembly are dependent on sampling depth and suggest that caution is required when interpreting diversity patterns from samples that integrate across depths. This is the first study focusing on assemblages of fungal communities in different soil layers on a relatively large scale, and we thus believe that this study is of great importance to researchers and readers in microbial ecology, especially in microbial biogeography, because the results can provide sampling guidance in future studies of microbial biogeography.

## Abstract (precis)

In this work, Illumina MiSeq amplicon sequencing of the ITS region was used to investigate the spatial variation and assembly mechanisms of fungal communities from different soil layers across paddy fields in subtropical China, and the results demonstrate the decreasing importance of environmental filtering and an increase in the importance of dispersal limitation in structuring fungal communities from shallower to deeper soils. Therefore, the results of this study highlight that perceived drivers of fungal community assembly are dependent on sampling depth and suggest that caution is required when interpreting diversity patterns from samples that integrate across depths. This is the first study focusing on assemblages of fungal communities in different soil layers on a relatively large scale, and we thus believe that this study is of great importance to researchers and readers in microbial ecology, especially in microbial biogeography, because the results can provide sampling guidance in future studies of microbial biogeography.

## INTRODUCTION

Understanding the spatial variation and the mechanisms regulating belowground microbial communities is essential for maintaining biodiversity (1, 2). Spatial variation in belowground bacterial communities has been well documented, but this is less extensively examined in soil fungal communities and especially those from paddy ecosystems. Paddy ecosystems, composing the third largest cropland area and the largest anthropogenic wetland on Earth, are crucial for global food security and environmental sustainability (3–5). In terrestrial ecosystems, fungi are prominent drivers of almost all terrestrial ecosystem functions such as decomposing organic plant material (6–8), with direct consequences for global carbon and nutrient dynamics (9–11). Currently, there is increasing awareness that the spatial variation in, and assemblages of, soil microbes may have important aboveground consequences, such as in plant community structure and ecosystem functioning (12, 13). Thus, more knowledge about spatial variation and the underlying ecological mechanisms governing fungal communities from paddy soils is required to support future predictions of ecosystem functioning in these soils.

Diversity patterns, especially beta-diversity patterns, provide evidence for the processes underlying community assembly (14). A distance-decay relationship (DDR) describes the negative relationship between community similarity and geographical distance (including both horizontal and vertical distance), which is considered to be one of the most common patterns in ecological communities (15, 16). DDR results from environmental filtering, dispersal limitation, and ecological drift, acting either in isolation or together. Low environmental heterogeneity, high dispersal rates, and ecological drift can homogenize the community, thus weakening DDRs (1, 14). Conversely, high environmental heterogeneity, increased dispersal limitation, and reduced ecological drift would enhance DDRs (1, 14). Some studies of fungi observed strong DDR between fungal community similarity and geographical distance across strong environmental gradients at fine scales (17, 18). At larger spatial scales, however, differences in the extent and spatial scaling of environmental heterogeneity, soil types, and host groups causes DDR to be observed (5), but not consistently (19).

DDRs can also provide insight into the driving factors of spatial variation in microbial communities and thus the processes that govern microbial community assembly. There are two different but complementary paradigms describing the assemblages of microbial communities, namely, niche-based and neutral-based models (20, 21). Niche-based theory posits that deterministic processes, including selection (variation selection and homogeneous selection) and niche partitioning are primarily controlling community assembly via differentiated habitat preferences and fitness of taxa (22). Neutral-based theory posits that stochastic processes, including those associated with dispersal properties, and random fluctuations in species abundances, in other words, ecological drift, are dominant in governing community assembly (22). Many communities are jointly regulated by both niche-based and neutral-based processes with different relative effects depending on climate, edaphic characteristics, spatial factors, biotic interactions, and biological activities (10, 23, 24), and the contributions of these factors to community assembly vary across organisms, host types, topography, sampling scale, and so on, which would also result in different DDR patterns (25, 26).

Much of our knowledge about terrestrial microbial ecology, including, but not limited to, DDR is from the top 20 cm of the soil column (27, 28). Yet, distinct microbial community structures have been observed between the topsoil and subsoil because of their different environments (29, 30). Soil properties change markedly with soil depth, especially oxygen content, and soil nutrients, including organic matter, nitrogen, and phosphorus, which all decline sharply. For example, oxygen can be detected only in the top 10 cm of nonflooded paddy soils; it is undetectable in deeper soils (31, 32). Soil organic matter also decreases with depth in nonflooded paddy soils, with values of 9.8 g kg^−1^ in topsoil, but undetectable levels at 100-cm depth. A similar pattern is also observed for total nitrogen, with 0.9 g kg^−1^ in topsoil and again undetectable levels at 100-cm depth (32). These differences in soil properties across different depths of soil may lead to stronger environmental filtering or nutrition competition (33) and lead to depth-dependent structuring of microbial communities and assemblages. However, rarely has research been conducted to investigate the microbial biogeographical and community patterns of various soil layers across large spatial scales, and certainly not in paddy soils.

If the prevalence of environmental filters, competitive interactions for resources, or barriers to dispersal vary across soil layers, DDRs calculated independently from each layer are likely to be distinct, reflecting changes in the relative importance of environmental factors or more neutral processes shaping the fungal community at different depths. Yet, this is rarely tested. Thus, we investigated the spatial variation and assembly mechanisms of fungal communities from different soil layers across paddy fields in subtropical China. We quantified a range of soil physiochemical properties to reflect environmental heterogeneity and used multiple statistical approaches to disentangle how fungal community assembles in different soil layers. We made the following predictions. (i) The form of DDRs describing how fungal community composition changes with increasing geographic distance between samples is distinct for each of the different soil layers. (ii) The relative importance of environmental factors and spatial factors (reflecting niche-based and more neutral-based mechanisms, respectively) in regulating fungal community assembly changes from shallow to deeper soil layers.

## RESULTS

### Soil properties.

**FIG 1:**
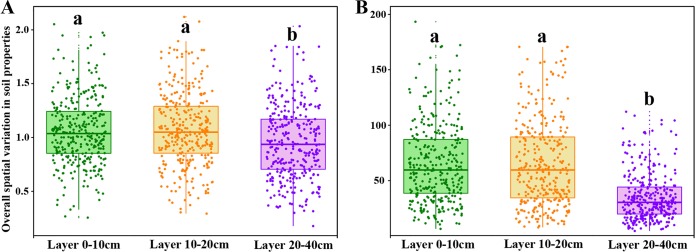
Boxplot showing the overall spatial variation in soil properties according to variance-covariance matrices based on all standardized soil properties (A) and original soil properties (B). Lowercase letters above the boxes indicate significant difference at *P* < 0.05.

All soil properties except total potassium (TK) exhibited considerable vertical variation (Table 1). Soil organic carbon (SOC), total nitrogen (TN), available nitrogen (AN), carbon-to-nitrogen (C/N) ratio, total phosphorus (TP), available phosphorus (AP), and cation exchange capacity (CEC) decreased significantly with soil depth, while pH and Fe showed the opposite tendency. Soil properties also varied widely across sampling sites in each soil layer (Table 1), while the average coefficient of variation showed no significant difference among soil layers (*P* > 0.05; see Table S1A in the supplemental material). Environmental variation (variance-covariance matrix) of standardized soil properties in the 0-to-10-cm (0-10cm)- and 10-20cm-deep layers was significantly higher than that in subsoil (Fig. 1A). Variance-covariance matrix based on all original soil properties confirmed this result (Fig. 1B). To examine how soil properties varied with geographic distance between samples of the same soil depths, we used Mantel correlation tests, and these tests showed that only a few soil properties in the 0-10cm-deep (AP and Fe) and 10-20cm-deep (C/N ratio and Fe) soils covaried with geographical distance. However, many soil properties, including SOC, TN, C/N ratio, AN, Fe, and pH covaried with geographical distance in the 20-40cm-deep soil layer (Table S1A). Additionally, soil parent material did not significantly influence soil properties (Table S1B).

**TABLE 1:** Soil properties in different soil layers

Soil property^*a*^ (unit)	0-10cm layer^*b*^				10-20cm layer				20-40cm layer			
	Max	Min	Mean	CV (%)	Max	Min	Mean	CV (%)	Max	Min	Mean	CV (%)
SOC (g/kg)	41.20	17.01	27.26 A	24.55	32.55	12.22	20.28 B	30.70	15.59	3.04	6.47 C	44.97
TN (g/kg)	4.74	1.77	2.93 A	26.25	3.85	1.29	2.19 B	32.11	1.94	0.32	0.78 C	49.00
TP (g/kg)	1.17	0.42	0.78 A	23.72	1.37	0.38	0.63 B	31.06	1.02	0.20	0.42 C	42.57
C/N ratio	12.66	9.11	10.94 A	7.47	12.82	8.73	10.86 A	8.71	14.55	7.19	10.01 B	18.33
TK (g/kg)	27.87	6.50	14.84 A	46.81	28.13	6.44	15.31 A	45.66	29.14	6.07	15.44 A	47.42
AN (mg/kg)	338.10	150.68	241.70 A	19.63	290.33	121.28	185.59 B	27.47	147.00	36.75	74.91 C	35.73
AP (mg/kg)	97.00	7.75	40.54 A	47.87	61.55	4.53	24.34 B	51.52	35.14	2.20	7.45 C	89.09
Fe (g/kg)	14.64	2.08	7.21 C	46.43	20.58	1.70	10.88 B	48.16	27.32	1.01	16.63 A	35.14
pH	5.49	4.71	5.11 B	3.90	5.53	4.75	5.10 B	3.42	6.52	4.93	5.53 A	5.82
CEC (mol/g)	18.75	8.14	11.83 A	23.85	14.67	7.14	10.32 B	19.56	13.17	6.73	9.31 B	19.92

a SOC, soil organic carbon; TN, total nitrogen; TP, total phosphorus; C/N ratio, carbon-to-nitrogen ratio; TK, total potassium; AN, available nitrogen; AP, available phosphorus; CEC, cation exchange capacity.

b Soil properties are shown for three different soil layers, 0 to 10 cm deep (0-10cm), 10-20cm, and 20-40cm. The maximum value (Max), minimum value (Min), mean value (n = 26) (Mean), and coefficient of variation (CV) are shown. Different letters in the Mean columns indicate significant differences among soil layers at *P* < 0.05.

10.1128/mSystems.00704-19.6 TABLE S1 Average coefficient of variation (CV) of soil properties in different soil layers, the correlations between soil properties and geographical distance in each soil layer, and the difference of soil properties between soil parent materials. Download Table S1, PDF file, 0.4 MB. Copyright © 2020 Li et al. 2020 Li et al. This content is distributed under the terms of the Creative Commons Attribution 4.0 International license.

### Overall structure of fungal communities.

Generally, *Ascomycota* (46.45%), *Zygomycota* (31.58%), and *Basidiomycota* (14.35%) were highly dominant across all samples (see Fig. S2 in the supplemental material). The relative abundance of *Ascomycota*, *Basidiomycota*, and *Chytridiomycota* tended to decrease with soil depth, while *Zygomycota* showed the opposite trend (Fig. S3). The relative abundance of *Glomeromycota* and *Neocallimastigomycota* showed no significant differences across different soil layers (Fig. S3). The relative abundance of *Ascomycota* and *Chytridiomycota* was significantly positively correlated with SOC, TN, C/N ratio, and AN but negatively correlated with Fe (*P* < 0.05; Table S2). The relative abundance of *Zygomycota* was negatively correlated with SOC, TN, CN, and AN but positively correlated with Fe (*P* < 0.05; Table S2). The relative abundance of *Basidiomycota* was significantly correlated only with AP (*r* = 0.387, *P* < 0.001) and Fe (*r* = −0.415, *P* < 0.001), and the relative abundance of *Glomeromycota* and *Neocallimastigomycota* was not significantly correlated with any soil properties (Table S2).

10.1128/mSystems.00704-19.7 TABLE S2 Correlations between relative abundance of fungal phyla and soil properties. Coefficients were determined by Pearson test. Download Table S2, PDF file, 0.4 MB. Copyright © 2020 Li et al. 2020 Li et al. This content is distributed under the terms of the Creative Commons Attribution 4.0 International license.

**FIG 2:**
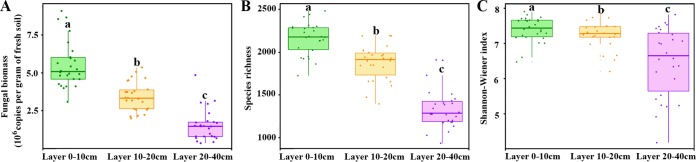
Boxplots showing the fungal biomass (A) and alpha-diversity of fungal communities (B and C) in each layer. Lowercase letters above the boxes indicate significant difference at *P* < 0.05.

**FIG 3:**
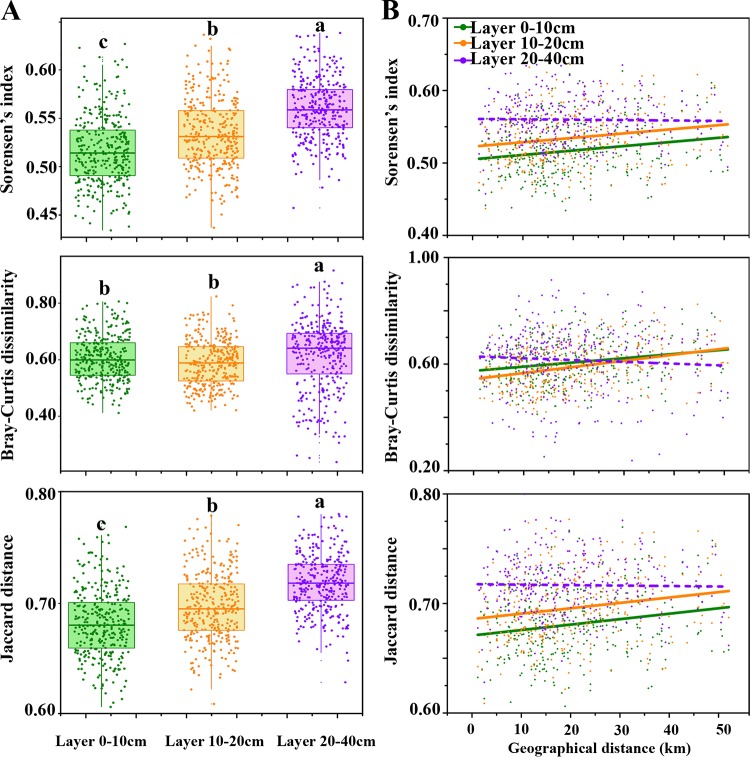
Beta-diversity of fungal communities. (A) Boxplots showing the values of beta-diversity; (B) distance-decay relationships between fungal community and geographical distance between samples. Lowercase letters above the boxes in panel A indicate significant difference at *P* < 0.05. Solid and dashed lines in panel B represent significant and nonsignificant relationships, respectively.

Fungal biomass and alpha-diversity (richness and Shannon-Wiener index) decreased significantly with soil depth (Fig. 2), whereas spatial variation in fungal biomass and alpha-diversity increased with soil depth (biomass, 27.98%, 27.64%, and 68.15%; average coefficients of richness, 8.73%, 11.03%, and 17.66%; average coefficients of Shannon-Wiener index, 4.81%, 5.67%, and 15.22%; all quoted for 0-10cm-, 10-20cm-, and 20-40cm-deep soil layers, respectively). Spatial variation in fungal community structure (i.e., Sørensen’s index, pairwise Bray-Curtis dissimilarity, and Jaccard distance) also increased significantly along with soil depth (Fig. 3A). Principal-coordinate analysis (PCoA) based on beta-diversity indices showed that samples cluster within each soil layer (Fig. S4), and one-way permutational multivariate analysis of variance (permANOVA) confirmed that the fungal communities in different soil layers were significantly different from each other (Table S3A). In addition to soil depth, soil parent material also significantly affected fungal community composition (Table S3B).

10.1128/mSystems.00704-19.8 TABLE S3 One-way permANOVA showing the difference of fungal communities between soil layers and between soil parent materials. Download Table S3, PDF file, 0.4 MB. Copyright © 2020 Li et al. 2020 Li et al. This content is distributed under the terms of the Creative Commons Attribution 4.0 International license.

### Correlation between fungal community and soil variables and geographical distance.

Partial Mantel tests showed that fungal beta-diversity indices were significantly correlated with more soil properties in shallower soil layers (0-10cm and 10-20cm), while only a few soil properties showed significant correlations with beta-diversity indices in the 20-40cm-deep layer (Table S4). We observed significant correlations between fungal beta-diversity indices and geographical distance between samples in both the 0-10cm and 10-20cm-deep soil layers (Fig. 3B). However, in the 20-40cm layer, no significant DDR could be observed regardless of the beta-diversity index used (Fig. 3B). In addition, between the 0-10cm and 10-20cm soil layers, the DDR slopes showed no significant difference across beta-diversity indices (*P* > 0.05).

10.1128/mSystems.00704-19.9 TABLE S4 Partial Mantel tests showing the correlations between soil properties and beta-diversity indexes of fungal communities. Download Table S4, PDF file, 0.4 MB. Copyright © 2020 Li et al. 2020 Li et al. This content is distributed under the terms of the Creative Commons Attribution 4.0 International license.

### Contributions of environmental and spatial factors to variation in fungal communities.

On the basis of three beta-diversity indices, forward selection procedures were respectively applied to select subsets of environmental and spatial variables which had significant effects on species composition (Table 2). Indices in the 0-10cm soil layer, regardless of which beta-diversity index was used, the environmental factors pH and TP, and spatial factor PCNM2 (principal coordinate 2 of neighbor matrices) were always selected. In the 10-20cm layer, the environmental factor Fe and the spatial factor PCNM2 could also be frequently selected. In the 20-40cm layer, when using Sørensen’s index and Jaccard distance, the environmental factors pH and TN and the spatial factors PCNM2, PCNM11, and PCNM3 were selected. If using Bray-Curtis dissimilarity, no environmental factor could be selected, while the spatial factor PCNM20 had significant effects on these two indices.

**TABLE 2:** Results of forward selection of environmental and spatial variables for fungal communities based on four beta-diversity indices^*a*^

Beta-diversity index	Variable source	0-10cm layer		10-20cm layer		20-40cm layer	
		Variable(s)	*R*^2^	Variable(s)	*R*^2^	Variable(s)	*R*^2^
Sørensen’s index	Environmental	pH, TP	0.118	Fe, pH	0.129	pH, TN	0.108
	Spatial	PCNM2, PCNM11	0.144	PCNM2, PCNM11, PCNM1	0.195	PCNM2, PCNM11, PCNM3	0.153
Bray-Curtis dissimilarity	Environmental	pH, TP	0.098	Fe	0.101	NS	NS
	Spatial	PCNM2	0.190	PCNM2, PCNM11, PCNM8	0.340	PCNM20	0.097
Jaccard distance	Environmental	pH, TP	0.119	Fe, pH	0.111	pH, TN	0.098
	Spatial	PCNM2, PCNM11	0.105	PCNM2, PCNM11, PCNM1	0.167	PCNM2, PCNM11, PCNM3	0.144

a Spatial variables were derived from vertical spatial coordinates using Moran’s eigenvector maps. NS, not statistically significant (*P* > 0.05).

Variation partitioning was applied based on the results of forward selection, and in general, the variation in species composition was largely (at least 81.94%) unexplained (Table 3). Of the variance that could be explained, spatial factors rather than environmental factors explained more across all soil layers, except in the 0-10cm soil layer where environmental variables explained slightly more. In the 20-40cm layer, environmental factors barely explain any variance (Table 3).

**TABLE 3:** Variation partitioning of fungal communities based on three beta-diversity indices^*a*^

Beta-diversity index and component	Variation explained (%)		
	0-10cm layer	10-20cm layer	20-40cm layer
Sørensen’s index			
[E|S]	1.47	4.26**	1.32
[S|E]	4.06**	6.58**	2.53*
[E∩S]	0.01	1.04	1.68
[R]	94.46	88.12	94.47
			
Bray-Curtis dissimilarity			
[E|S]	6.39**	0.74	NS
[S|E]	3.41**	12.97***	NS
[E∩S]	1.51	4.35	NS
[R]	88.69	81.94	NS

Jaccard distance			
[E|S]	3.21**	2.89**	1.01
[S|E]	2.20**	4.52**	1.82*
[E∩S]	0.44	0.63	1.08
[R]	94.15	91.95	96.09

a Four different components are shown: pure environmental fraction ([E|S]), pure spatial fraction ([S|E]), shared fraction of environmental and spatial effects ([E∩S]), unexplained fraction ([R]). Values shown in boldface type showed significant effects. *, **, and *** indicate significant effects at *P* < 0.05, 0.01, and 0.001, respectively. NS indicates that variation partitioning could not be conducted because the subset of environmental variable was lacking.

### Community assembly process measurements with dominance test and normalized stochasticity ratio (NST).

**FIG 4:**
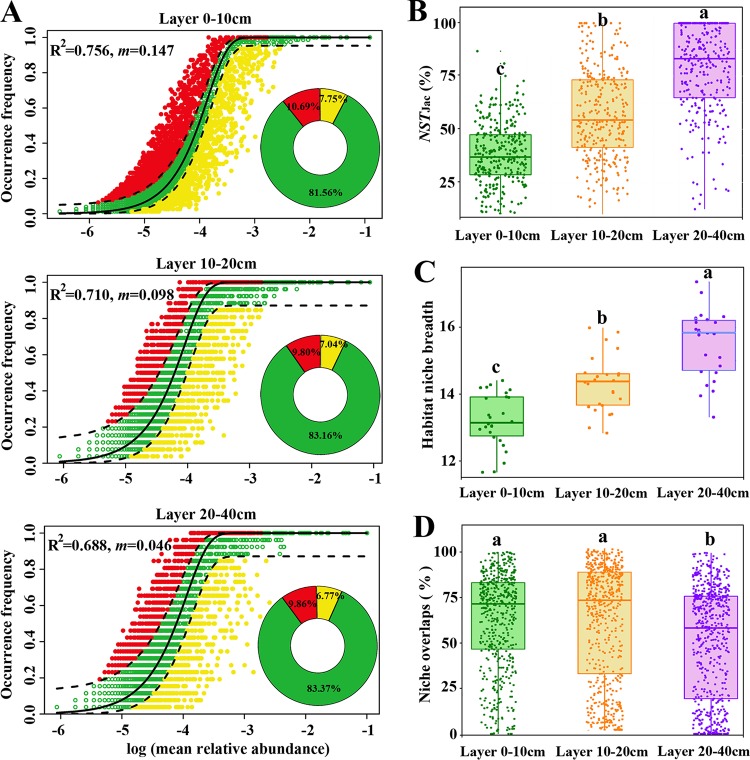
Community assembly process measurements by the dominance test (A), normalized stochasticity ratio (NST) index (B), habitat niche breadth (C), and niche overlaps (D). (A) OTUs that occur more frequently than predicted by the model are shown in red, while those that occur less frequently than predicted are shown in yellow. OTUs that occur within prediction are shown in green. Dashed lines represent 95% confidence intervals around the model prediction (black line). (B) The normalized stochasticity test (NST) was developed based on Jaccard distance (NST_jac_) with 50% as the boundary point between more deterministic (<50%) and more stochastic (>50%) assembly. (C and D) One-way ANOVA and nonparametric Mann-Whitney *U* test were conducted to test the significance of difference in habitat niche breadth (C) and niche overlaps (D), respectively.

Based on *R*^2^ values (0.756, 0.710, and 0.688 for 0-10cm-, 10-20cm-, and 20-40cm-deep soil layers, respectively) and the proportions of outlying taxa beyond the dashed line (18.44%, 16.84%, and 16.64% for 0-10cm-, 10-20cm-, and 20-40cm-deep soil layers, respectively) reflecting those outside model predictions, the dominance test showed that fungal community assemblages of each soil layer were well described by neutral-based models (Fig. 4A). Operational taxonomic units (OTUs) outside model predictions accounted for, on average, 77.28%, 22.46%, and 28.85% of total sequences in 0-10cm-, 10-20cm-, and 20-40cm-deep soil layers, respectively. The random forest model showed that these OTUs were more influenced by environmental factors than those inside model predictions (Table S5). The *m* value (migration rate, 0.147, 0.098, and 0.046 for 0-10cm-, 10-20cm-, and 20-40cm-deep soil layers, respectively) tended to decrease along with soil depth, suggesting that fungi in soil layer 0-10cm were highly diffused.

10.1128/mSystems.00704-19.10 TABLE S5 Random forest model showing the potential important factors influencing OTUs that occurred outside or inside predictions by dominance test. Download Table S5, PDF file, 0.5 MB. Copyright © 2020 Li et al. 2020 Li et al. This content is distributed under the terms of the Creative Commons Attribution 4.0 International license.

The NST based on Jaccard distance (NST_jac_) index showed that fungi within the 0-10cm soil layer were predominately governed by deterministic processes (NST_jac_ = 37.74%), but fungi within the 20-40cm soil layer were primarily controlled by stochastic processes (NST_jac_ = 70.41%). Within the 10-20cm soil layer, determinism played a marginally stronger role in controlling fungal community assemblages (*NST*_jac_ = 57.40%; Fig. 4B). These observations suggested that deterministic processes decreased while stochastic processes increased with soil depth. Supporting results from NST_jac_, NST based on Bray-Curtis dissimilarity (NST_bray_) also gradually increased along with soil depth (Fig. S5; 36.14%, 52.66%, and 76.35% for 0-10cm-, 10-20cm-, and 20-40cm-deep soil layers, respectively).

Community-level habitat niche breadths (*B*com) were estimated, and fungi in the 20-40cm soil layer showed the highest values, followed by those from the 10-20cm layer and finally the 0-10cm layer (Fig. 4C). In contrast to *B*com, the niche overlaps among fungi were lowest in the deepest soil layer (20-40cm), while fungi in the shallower layers showed no significant difference in niche overlaps (Fig. 4D).

## DISCUSSION

This study quantified spatial variation and drivers of fungal community assembly in paddy field soils from a typical region of subtropical China. Our results consistently revealed that spatial variation in fungi was higher in topsoil than in subsoil and demonstrated the decrease in importance of environmental filtering and an increase in the importance of dispersal limitation in structuring fungal communities from shallower to deeper soils.

We observed obvious spatial variation in fungal communities among our sampling sites (Fig. 3); while the spatial variation was lower than reported from studies conducted on very large scales (34, 35), it was higher than that recorded at smaller scales (36, 37), suggesting that the degree of spatial variation observed in fungal communities was dependent on the scale. Distance-decay relationships revealed significant correlations between changes in fungal community composition and the geographical distance between samples for 0-10cm and 10-20cm soil layers, but not in the deeper 20-40cm layer (Fig. 3B), suggesting that DDR occurred only in topsoil. Additionally, the slopes of the DDRs from the 0-10cm and 10-20cm soil layers were similar, which may result from the uniform tillage operations that homogenize topsoil to some extent. It should be noted that some factors, such as low environmental heterogeneity, high dispersal, and ecological drift, would greatly weaken the DDR via homogenizing communities (38). Environmental heterogeneity was significantly higher in the shallower soil layers (0-10cm and 10-20cm) than in deeper soil layers, suggesting that low environmental heterogeneity indeed weakened the DDR in the 20-40cm soil layer. Although shallower soil layers (0-10cm and 10-20cm) had higher dispersal rate than deeper soil layers (Fig. 4A), the relatively higher environmental heterogeneity maintained a significant DDR. Judging from DDR and migration rate, we postulate that the fungal communities in shallower soils (0-10cm and 10-20cm deep) are relatively more influenced by environmental factors and dispersal than deeper soils. Additionally, partial Mantel tests showed that a greater number of soil properties were correlated with fungal community composition in shallower soil layers than deeper soil layers (see Table S4 in the supplemental material), whereas few of these soil properties were correlated with geographical distance in these layers (Table S1). This implies that fungal communities in shallower layers are influenced by nonspatially autocorrelated environmental factors.

While higher environmental heterogeneity leads to higher structural heterogeneity of communities (1, 14), our study results showed that the degree of environmental variability did not match the extent of community variability (Fig. 1 and 3A). Thus, environmental factors and environmental variability have less of an impact on variation in species composition compared with other nonenvironmental factors here. Both partial Mantel tests and forward selection demonstrated that some soil properties, especially pH, had significant effects on species composition (Table S4 and Table 2), but their effects were limited and unlikely to be ecologically meaningful. For example, soil pH is always demonstrated to be a key factor affecting microbial assemblages (39), while the coefficients of variation in pH are never higher than 6% in our research, which can keep the soils under relatively strong acidity.

Variation partitioning showed that environmental factors played a slightly more dominant role in driving fungal community assembly in the 0-10cm soil layer. However, spatial factors, rather than environmental factors, played a far larger and more important role in governing fungal community assembly in the deeper soil layers (10-20cm and 20-40cm) (Table 3), reflecting an increased importance of neutral processes with increasing soil depth. Some other studies on paddy soils found that spatial factors better predicted for fungal community composition compared with environmental factors (5), in contrast to studies of forest soils where environmental factors are generally shown as the better predictors (10). However, our results show that the relative importance of environmental versus spatial factors is soil depth dependent, as environmental factors can better predict fungal communities in the 0-10cm-deep paddy soil layer, while spatial factors are better predictors in deeper soil layers (10-20cm and 20-40cm). Such a pattern should be closely related to the decreased environmental heterogeneity and dispersal rate with soil depth, exposing fungi in topsoil to higher environmental selection and immigration (40). However, a large proportion of variation remains unexplained whichever beta-diversity indices were used (at least 81.94%; Table 3). The unexplained variation may be attributed to unmeasured environmental variables, which could include total dissolved oxygen, the distance from the river or nearby streams, and drainage potential. For example, dissolved oxygen may vary greatly across different soil layers (31, 32), promoting redox gradients that may influence the variation in fungal community composition. The distance from sampling site to the nearest river or stream may be another factor affecting fungal community composition, as these bodies of water potentially help the free movement of fungi. In fact, we found it a common phenomenon that a large proportion of variation is rarely explained when using variation partitioning. For example, nearly 90% of variation cannot be explained by spatial or environmental factors on plant community (41), aquatic organisms (42), and soil eukaryotes (33). More importantly, although sampling effects or unmeasured variability may contribute to the unexplained variation, it is tempting to speculate that the high fraction of unexplained variance could be caused by the evolutionary noise produced by ecologically neutral processes of diversification, i.e., through random ecological drift, which cannot be determined by mathematical models (40, 43, 44).

Dominance test, NST index, and habitat niche breadth were used to help explain the spatial variation in fungal communities and their associated ecological drivers. The dominance test showed that the models had high *R*^2^ values, and more than 80% of species had frequencies within predicted ranges (Fig. 4A), suggesting that the frequency with which fungi occurred in different soil layers can be well described by the neutral model. Even so, some nonneutral process should also be considered. Within each soil layer, there were some fungal species, less than 20%, whose distributions deviated from neutral predictions (Fig. 4A). For example, in the 0-10cm soil layer, the <20% OTUs occurring outside predictions accounted on average for >77% of total sequences, while in 10-20cm and 20-40cm soil layers, the <20% OTUs occurring outside predictions accounted for < 23% of total sequences on average (Fig. 4A). Random forest models showed that OTUs outside model predictions were more influenced by environmental factors than those inside model predictions (Table S5).These results suggested that more fungi (higher relative abundance, rather than a larger number of taxa) in the 0-10cm layer were selectively enriched or excluded as a result of environmental selection (45). The model also showed a very high migration rate (*m*) in topsoil and a very low migration rate in subsoil, implying high and unhindered dispersal in topsoil and significant dispersal limitation in subsoil. While some studies suggest that fungi are free to disperse, and thus dispersal limitation does not exist (46), other studies suggest that as fungi are relatively large compared with other microbes (e.g., bacteria) (47), their dispersal may be limited. In our study, the migration rate of fungi was much higher in topsoil. This is easy to monitor, because paddy fields are covered with water for most of the year, and the flow of water would greatly help the free movement of fungi. However, the fungi in subsoil can hardly disperse prior to moving from subsoil to topsoil or, at least, can hardly widely disperse. Additionally, tillage operations that may contribute to the dispersal of fungi are typically only carried out in the topsoil.

The NST index suggested that the relative importance of deterministic processes over stochastic processes in structuring fungal communities decreased with soil depth (Fig. 4B and Fig. S5). The higher environmental heterogeneity in the topsoil exposed soil fungi to a greater range of environmental filters, which drives the unambiguously deterministic process of environmental selection (48). Thus, our results imply that as soil depth increases, environmental selection has an ever lower influence on structuring fungal communities. In contrast, the relative influence of stochastic process in structuring fungal communities increased with soil depth (NST), and this is likely related to increased dispersal limitation (22). Although some stochastic processes such as diversification and ecological drift, which are problematic to quantify, may also increase along with soil depth.

Species with wider niche breadth are considered to be generalists which are less influenced by environmental factors because of higher environmental tolerances (49, 50). In our study, habitat niche breadth of fungi continuously increased along with soil depth (Fig. 4C), suggesting that fungi in subsoil with wider niche breadth were governed less by environmental filtering. We initially expected that niche overlaps should be higher in subsoil because of relatively lower resources. However, niche overlaps among fungi were significantly lower in the 20-40cm soil layer than in the 0-10cm and 10-20cm layers (Fig. 4D). This is likely a result of lower fungal biomass and richness (Fig. 2). Fungi in the 20-40cm layer occupied wider niche breadths with lower niche overlaps, suggesting that they can effectively utilize an array of resources with less competition (51) and that they are better adapted to the local environment. Thus, the fungi in the 20-40cm soil layer should be less influenced by deterministic processes, including environmental filtering and competitive exclusion.

### Conclusions.

We observed obvious spatial variation in fungal communities of paddy fields in subtropical China and found that environmental heterogeneity decreased along with soil depth, while spatial variation in fungal communities showed the opposite tendency. An array of statistical analyses revealed that the fungal community assembly in the 0-10cm-deep layer was primarily governed by environmental filtering and high dispersal, while in the deeper layer (20-40cm), it was primarily governed by dispersal limitation and minimal environmental filtering. Both environmental filtering and dispersal limitation controlled the fungal community assembly in the 10-20cm-deep layer, with dispersal limitation playing the major role. This work highlights that perceived drivers of fungal community assembly are dependent on sampling depth. Thus, future studies interpreting diversity patterns from soil samples that integrate over a wide range of depths should do so with caution, as different ecological mechanisms are likely acting in different soil layers.

## MATERIALS AND METHODS

### Soil sampling and physicochemical characterization.

Soil samples were collected near the end of December 2017 from red paddy soils in Yujiang (Jiangxi Province, China; 116°41′ E to 117°09′ E, and 28°04′ N to 28°37′ N), where > 85% of cultivated land is paddy fields. Sampling sites have subtropical monsoon climates, with abundant sunshine and rainfall (mean annual sunshine hours, 1,739.4 h; mean annual temperature, 17.6°C;mean annual precipitation, 1,750 mm). The total sampling area is 927 km^2^, including 78.2% of hills and 21.2% of plains. The cropping system here is mainly double cropping rice (Oryza sativa L.) (i.e., early and late season rice). Rotary tillage to a depth of 15 to 20 cm (15-20cm) is conducted prior to seedling. The natural conditions, including climate, soil properties, topography, geomorphology, cropping system, and social and economic conditions, including productivity level, are typically representative of subtropical areas of southern China (52).

**FIG 5:**
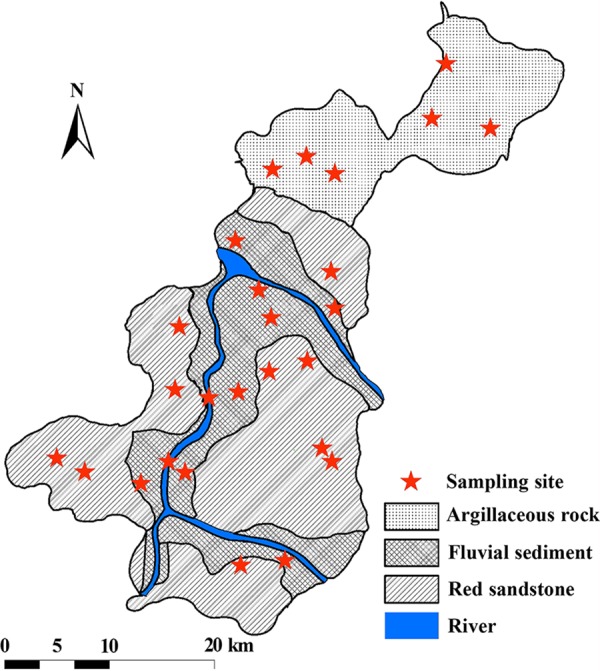
Locations of the sampling sites.

Sampling sites were chosen to satisfy the following conditions. (i) The whole region needed to be covered. (ii) The main parent material of the soils needed to be included. (iii) Field management, including cropping system and fertilizer applications, should be uniform. On the basis of these principles, 26 sites were selected, with pairwise geographical distances ranging from 1.3 km to 50.7 km (Fig. 5; see Fig. S1 in the supplemental material). The soil samples were collected in December 2017 after the harvest and in the absence of water flooding. Within each site, five 40-cm-deep soil cores (6-cm diameter, free from rice roots) were collected at random locations and partitioned into three depth intervals: 0-10cm, 10-20cm, and 20-40cm. Samples were refrigerated at 4°C using a portable fridge and transported to the laboratory. Samples from each plot were composited by depth, homogenized, and subsampled for subsequent analyses. Subsamples for physical and chemical properties were air dried, ground, and sieved through 2-mm mesh. Subsamples for microbial properties were stored at −40°C.

10.1128/mSystems.00704-19.1 FIG S1 Locations of sampling sites. The number of each site is corresponded with the sample number of the sequences that we submitted to NCBI Sequence Read Archive (SRA) under the accession number SRP200912. Download FIG S1, PDF file, 0.9 MB. Copyright © 2020 Li et al. 2020 Li et al. This content is distributed under the terms of the Creative Commons Attribution 4.0 International license.

10.1128/mSystems.00704-19.2 FIG S2 Species composition of fungal communities at the phylum level. Download FIG S2, PDF file, 0.1 MB. Copyright © 2020 Li et al. 2020 Li et al. This content is distributed under the terms of the Creative Commons Attribution 4.0 International license.

10.1128/mSystems.00704-19.3 FIG S3 Relative abundance of each fungal phylum. Different letters indicate significant differences at *P* < 0.05. Download FIG S3, PDF file, 0.2 MB. Copyright © 2020 Li et al. 2020 Li et al. This content is distributed under the terms of the Creative Commons Attribution 4.0 International license.

10.1128/mSystems.00704-19.4 FIG S4 Principal-coordinate analysis (PCoA) based on three beta-diversity indexes. Download FIG S4, PDF file, 0.2 MB. Copyright © 2020 Li et al. 2020 Li et al. This content is distributed under the terms of the Creative Commons Attribution 4.0 International license.

10.1128/mSystems.00704-19.5 FIG S5 NST index based on Bray-Curtis dissimilarity showing fungal community assemblages. Different letters indicate significant differences at *P* < 0.05. Download FIG S5, PDF file, 0.7 MB. Copyright © 2020 Li et al. 2020 Li et al. This content is distributed under the terms of the Creative Commons Attribution 4.0 International license.

Soil chemical properties were determined using the methods described by Pansu and Gautheyrou (53). Soil pH was assayed using a pH meter (FE30; Mettler-Toledo) with 1:2.5 soil-water suspension. Cation exchange capacity (CEC) was determined by saturating the exchange sites of 1 g of each sample twice with 1 M ammonium acetate solution at pH 7, followed by replacing the adsorbed ammonium ions twice with 1 M KCl. Soil organic carbon (SOC) was titrated against 0.5 M ferrous iron solution after it had been digested with 0.8 M K_2_Cr_2_O_4_ and concentrated H_2_SO_4_ (vol/vol, 1:1) at 150°C for 30 min. Total nitrogen (TN) and available nitrogen (AN) were measured as Kjeldahl N. Briefly, the soil sample was heated and boiled with concentrated H_2_SO_4_. The total nitrogen (TN) was then absorbed by 2% boric acid solution and titrated against 0.1 M sulfuric acid. The available nitrogen was hydrolyzed by 1 M sodium hydroxide and measured by microdiffusion methods. Total phosphorus (TP) and available phosphorus (AP) were extracted with HF-HClO_4_ and sodium bicarbonate, respectively, and then determined by the molybdenum blue method using an UV spectrophotometer at 700 nm. Total potassium (TK) was determined using flame emission spectrometry after the soil had been digested in concentrated HF-HClO_4_ (vol/vol, 2:1). Free iron (Fe) of the soil was extracted by dithionite-citrate–bicarbonate (DCB) solution with 1 g of soil being digested in 40 ml of 0.3 M sodium citrate and 5 ml of 1 M sodium hydrogen carbonate at 353 K for 30 min, and the amount of free iron was then determined by flame atomic absorption spectrophotometry.

### Soil DNA extraction, amplification, Illumina sequencing, and sequence processing.

Soil DNA was extracted from 0.5 g of soil (fresh weight) using a FastDNA SPIN kit (MP Biomedicals, CA, USA) and then subsequently purified using a PowerClean DNA clean-up kit (MoBio, CA, USA) according to the manufacturers’ instructions. The concentration and quality of the extracted DNA were measured using a NanoDrop ND-1000 spectrophotometer (NanoDrop Technologies, DE, USA). Quantitative PCR was done on the Bio-Rad CFX96 touch real-time PCR detection system following protocols previously described (54). Results are reported as gene copy numbers and are used to reflect fungal biomass.

Each of the 78 DNA samples was amplified separately using the fungal PCR primers ITS1F (5′-CTTGGTCATTTAGAGGAAGTAA-3′) and ITS2 (5′-GCTGCGTTCTTCATCGATGC-3′) (55) that target the internal transcribed spacer 1 (ITS1) region. PCR products were then sequenced on the Illumina MiSeq PE250 platform. Raw sequence data were analyzed using the Quantitative Insights into Microbial Ecology (QIIME) pipeline (v1.9.1) (http://qiime.org/) (56). Paired-end reads were merged using FLASH (57). Reads with length of <200 bp or with average quality scores of <25 were removed, resulting in 796,863 high-quality sequences. ITSx 1.0.11 (http://microbiology.se/software/) was then used to remove 5.8S and 28S regions from merged sequences (58). Any chimeric sequences were removed using the USEARCH tool based on the UCHIME algorithm (59). Operational taxonomic unit (OTU) picking was performed using the “*pick_otus.py*” command with the nondefault UCLUST algorithm (the parameters were as follows: picking method, uclust; similarity of 0.97; max_accepts of 20, and max_rejects of 100) (60). Sequences were clustered into 12,474 OTUs after excluding singletons and rarefying to 44,296 sequences per sample (based on the sample with the minimum numbers of reads) (61, 62). The taxonomic identity of each OTU was then determined based on comparisons against the UNITE database (v7) (https://unite.ut.ee/).

### Statistical analysis.

Alpha-diversity indices, including richness and Shannon-Wiener index were calculated in QIIME using the “*alpha_diversity.py*” script. Statistically significant differences in soil properties, fungal biomass, and alpha-diversity indices were determined by one-way analysis of variance (ANOVA), along with the use of Duncan’s test for multiple comparisons (*P* < 0.05). If the variances of observations were heterogeneous, nonparametric Mann-Whitney *U* test were used to determine the statistical significance. Variance-covariance matrix based on all soil properties was calculated to indicate the overall variation in soil properties. Three beta-diversity indices, including Sørensen’s index, pairwise Bray-Curtis dissimilarity, and Jaccard distance coupled with principal-coordinate analysis (PCoA) were conducted to indicate the community dissimilarities. Partial Mantel tests were conducted to determine the potential effects of each soil property on fungal composition.

Variation-partitioning analysis was conducted to disentangle the relative importance of environmental factors and spatial factors on variation in fungal communities. Spatial variables were derived from the principal coordinates of neighbor matrices (PCNM) algorithm, which was able to deconvolute total spatial variation into a discrete set of explanatory spatial scales (63). Forward selection procedures were subsequently used to select respective subsets of environmental and spatial variables. The forward selection was stopped if the significance level (*P* > 0.05) was reached, or if no improvement of selection criterion (*R*^2^) was seen when adding any additional variables. A two-way permutational multivariate analysis of variance (permANOVA) was then performed with the selected variables using the R script provided by Wu et al. (38). Pure environmental variation without a spatial component represents the strength of environmental filtering, while pure spatial variation without an environmental component is interpreted as the effect of dispersal limitation. The fractions of explained variance are based on adjusted fractions (*R*^2^_adj_, adjusted coefficient of multiple determination), which accounts for the number of variables and sample sizes. The significance of each component via partitioning was evaluated with a permutation test, except for the interaction term and residuals (these cannot be tested statistically).

A neutral assembly model (the so-called dominance test) was used to determine the potential contribution of neutral processes to the community assembly by predicting the relationship between the occurrence frequency of OTUs and their relative abundance (64). This model evaluates whether the microbial assembly process from a metacommunity follows a neutral model (inside model predictions) or a niche-based process (outside model predictions) as a function of the metacommunity log abundance. Random forest analyses were subsequently performed to quantitatively evaluate the importance of predictors influencing OTUs that occurred outside or inside predictions of the dominance test. The importance of each predictor was determined by assessing the decrease in prediction accuracy (that is, the increase in the mean square error [MSE] between observations and predictions) when the data for the predictor were randomly permuted. This decrease was averaged over all trees to produce the final measure of importance. These analyses were conducted using the “*randomForest*” package of the R statistical language (65). The significance of predictor importance was assessed by using the “*rfPermute*” package.

We further applied the normalized stochasticity ratio (NST) to help confirm fungal community assembly processes. NST is an index developed with 50% as the boundary point between more deterministic (<50%) and more stochastic (>50%) assembly (66). We choose NST to indicate assembly processes because our research met the requirements of this method: (i) local/landscape scale sampling as opposed to global scale; (ii) *n *≥ 6. This analysis was conducted in the R statistical language (65) using “*NST*” package (the parameters were as follows: “dist.method” of “bray”/”jaccard,” “abundance.weighted” of “TRUE”, and “rand” of “1000”). By considering the overall performance of similarity metrics, NST based on Jaccard distance (NST_jac_) is recommended for estimating the magnitude of stochasticity in community assembly (66), but NST based on Bray-Curtis dissimilarity (NST_bray_) is also calculated in our research to further verify NST_jac_.

Niche breadth and niche overlaps were respectively calculated according to Levin’s niche breadth index and Levin’s niche overlap index (38). Briefly, Levin’s niche breadth index was determined as follows:

$$B_{j}=1/\overset{N}{\underset{i=1}{\sum }}P_{\mathrm{ij}}^{2}$$

where *B*_j_ represents the habitat niche breadth of OTU*_j_* in a metacommunity, *N* is the total number of communities of each metacommunity, and *P_ij_* is the proportion of OTU *j* in community *i*. A high *B* indicates that the OTU occurs widely and evenly along a wide range of locations, representing wide habitat niche breadth. We calculated the average *B* values from all taxa in a single community (*B*com) as an indicator of habitat niche breadth at the community level. Levins’ niche overlap index (*O*) was calculated as follows:

$$O_{\mathrm{jk}}=\overset{N}{\underset{i=1}{\sum }}(P_{\mathrm{ij}}P_{\mathrm{kj}})/\overset{N}{\underset{i=1}{\sum }}{\left(P_{\mathrm{ij}}\right)}^{2}$$

where *O_jk_* represents the niche overlap between OTU*_j_* and OTU*_k_*, *N* is the total number of communities of each metacommunity, *P_ij_* is the proportion of OTU *j* in community *i*, *P_kj_* is the proportion of OTU *k* in community *i*. A high *O* indicates that the species exhibited more niche overlap.

### Data accessibility.

The ITS sequences used in this study were submitted to the NCBI Sequence Read Archive (SRA) under the accession number SRP200912.

## References

[1] Nemergut DR, Schmidt SK, Fukami T, O’Neill SP, Bilinski TM, Stanish LF, Knelman JE, Darcy JL, Lynch RC, Wickey P, Ferrenberg S 2013 Patterns and processes of microbial community assembly. Microbiol Mol Biol Rev 77:342–356. doi:10.1128/MMBR.00051-12. 24006468 PMC3811611

[2] Pagaling E, Strathdee F, Spears BM, Cates ME, Allen RJ, Free A 2014 Community history affects the predictability of microbial ecosystem development. ISME J 8:19–30. doi:10.1038/ismej.2013.150. 23985743 PMC3869022

[3] Leff B, Ramankutty N, Foley JA 2004 Geographic distribution of major crops across the world. Global Biogeochem Cycles 18:GB1009. doi:10.1029/2003gb002108.

[4] Kogel-Knabner I, Amelung W, Cao ZH, Fiedler S, Frenzel P, Jahn R, Kalbitz K, Kolbl A, Schloter M 2010 Biogeochemistry of paddy soils. Geoderma 157:1–14. doi:10.1016/j.geoderma.2010.03.009.

[5] Yuan CL, Zhang LM, Hu HW, Wang JT, Shen JP, He JZ 2018 The biogeography of fungal communities in paddy soils is mainly driven by geographic distance. J Soils Sediments 18:1795–1805. doi:10.1007/s11368-018-1924-4.

[6] Crowther TW, Maynard DS, Crowther TR, Peccia J, Smith JR, Bradford MA 2014 Untangling the fungal niche: the trait-based approach. Front Microbiol 5:579. doi:10.3389/fmicb.2014.00579. 25400630 PMC4215788

[7] Treseder KK, Lennon JT 2015 Fungal traits that drive ecosystem dynamics on land. Microbiol Mol Biol Rev 79:243–262. doi:10.1128/MMBR.00001-15. 25971588 PMC4429240

[8] Maynard DS, Bradford MA, Covey KR, Lindner D, Glaeser J, Talbert DA, Tinker PJ, Walker DM, Crowther TW 2019 Consistent trade-offs in fungal trait expression across broad spatial scales. Nat Microbiol 4:846–853. doi:10.1038/s41564-019-0361-5. 30804547

[9] Nishizawa T, Zhaorigetu, Komatsuzaki M, Sato Y, Kaneko N, Ohta H 2010 Molecular characterization of fungal communities in non-tilled, cover-cropped upland rice field soils. Microbes Environ 25:204–210. doi:10.1264/jsme2.ME10108. 21597240

[10] Tedersoo L, Bahram M, Polme S, Koljalg U, Yorou NS, Wijesundera R, Ruiz LV, Vasco-Palacios AM, Thu PQ, Suija A, Smith ME, Sharp C, Saluveer E, Saitta A, Rosas M, Riit T, Ratkowsky D, Pritsch K, Poldmaa K, Piepenbring M, Phosri C, Peterson M, Parts K, Partel K, Otsing E, Nouhra E, Njouonkou AL, Nilsson RH, Morgado LN, Mayor J, May TW, Majuakim L, Lodge DJ, Lee SS, Larsson KH, Kohout P, Hosaka K, Hiiesalu I, Henkel TW, Harend H, Guo LD, Greslebin A, Grelet G, Geml J, Gates G, Dunstan W, Dunk C, Drenkhan R, Dearnaley J, De Kesel A, Dang T, 2014 Global diversity and geography of soil fungi. Science 346:1256688. doi:10.1126/science.1256688. 25430773

[11] Hu HW, Chen D, He JZ 2015 Microbial regulation of terrestrial nitrous oxide formation: understanding the biological pathways for prediction of emission rates. FEMS Microbiol Rev 39:729–749. doi:10.1093/femsre/fuv021. 25934121

[12] Berendsen RL, Pieterse CMJ, Bakker P 2012 The rhizosphere microbiome and plant health. Trends Plant Sci 17:478–486. doi:10.1016/j.tplants.2012.04.001. 22564542

[13] Fitzpatrick CR, Copeland J, Wang PW, Guttman DS, Kotanen PM, Johnson M 2018 Assembly and ecological function of the root microbiome across angiosperm plant species. Proc Natl Acad Sci U S A 115:E1157–E1165. doi:10.1073/pnas.1717617115. 29358405 PMC5819437

[14] Vellend M 2010 Conceptual synthesis in community ecology. Q Rev Biol 85:183–206. doi:10.1086/652373. 20565040

[15] Nekola JC, White PS 1999 The distance decay of similarity in biogeography and ecology. J Biogeogr 26:867–878. doi:10.1046/j.1365-2699.1999.00305.x.

[16] Poulin R 2003 The decay of similarity with geographical distance in parasite communities of vertebrate hosts. J Biogeogr 30:1609–1615. doi:10.1046/j.1365-2699.2003.00949.x.

[17] Lekberg Y, Koide RT, Rohr JR, Aldrich-Wolfe L, Morton JB 2007 Role of niche restrictions and dispersal in the composition of arbuscular mycorrhizal fungal communities. J Ecol 95:95–105. doi:10.1111/j.1365-2745.2006.01193.x.

[18] Dumbrell AJ, Nelson M, Helgason T, Dytham C, Fitter AH 2010 Relative roles of niche and neutral processes in structuring a soil microbial community. ISME J 4:337–345. doi:10.1038/ismej.2009.122. 19924158

[19] Xiao X, Liang YT, Zhou S, Zhuang SY, Sun B 2018 Fungal community reveals less dispersal limitation and potentially more connected network than that of bacteria in bamboo forest soils. Mol Ecol 27:550–563. doi:10.1111/mec.14428. 29134738

[20] Chase JM 2010 Stochastic community assembly causes higher biodiversity in more productive environments. Science 328:1388–1391. doi:10.1126/science.1187820. 20508088

[21] Rosindell J, Hubbell SP, Etienne RS 2011 The unified neutral theory of biodiversity and biogeography at age ten. Trends Ecol Evol 26:340–348. doi:10.1016/j.tree.2011.03.024. 21561679

[22] Zhou JZ, Ning DL 2017 Stochastic community assembly: does it matter in microbial ecology? Microbiol Mol Biol Rev 81:e00002-17. doi:10.1128/MMBR.00002-17. 29021219 PMC5706748

[23] Robinson CJ, Bohannan BJM, Young VB 2010 From structure to function: the ecology of host-associated microbial communities. Microbiol Mol Biol Rev 74:453. doi:10.1128/MMBR.00014-10. 20805407 PMC2937523

[24] Bahram M, Hildebrand F, Forslund SK, Anderson JL, Soudzilovskaia NA, Bodegom PM, Bengtsson-Palme J, Anslan S, Coelho LP, Harend H, Huerta-Cepas J, Medema MH, Maltz MR, Mundra S, Olsson PA, Pent M, Polme S, Sunagawa S, Ryberg M, Tedersoo L, Bork P 2018 Structure and function of the global topsoil microbiome. Nature 560:233. doi:10.1038/s41586-018-0386-6. 30069051

[25] Cottenie K 2005 Integrating environmental and spatial processes in ecological community dynamics. Ecol Lett 8:1175–1182. doi:10.1111/j.1461-0248.2005.00820.x. 21352441

[26] Padial AA, Ceschin F, Declerck SAJ, De Meester L, Bonecker CC, Lansac-Toha FA, Rodrigues L, Rodrigues LC, Train S, Velho LFM, Bini LM 2014 Dispersal ability determines the role of environmental, spatial and temporal drivers of metacommunity structure. PLoS One 9:e111227. doi:10.1371/journal.pone.0111227. 25340577 PMC4207762

[27] Fierer N, Schimel JP, Holden PA 2003 Variations in microbial community composition through two soil depth profiles. Soil Biol Biochem 35:167–176. doi:10.1016/S0038-0717(02)00251-1.

[28] Hartmann M, Lee S, Hallam SJ, Mohn WW 2009 Bacterial, archaeal and eukaryal community structures throughout soil horizons of harvested and naturally disturbed forest stands. Environ Microbiol 11:3045–3062. doi:10.1111/j.1462-2920.2009.02008.x. 19659501

[29] Eilers KG, Debenport S, Anderson S, Fierer N 2012 Digging deeper to find unique microbial communities: the strong effect of depth on the structure of bacterial and archaeal communities in soil. Soil Biol Biochem 50:58–65. doi:10.1016/j.soilbio.2012.03.011.

[30] Chu HY, Sun HB, Tripathi BM, Adams JM, Huang R, Zhang YJ, Shi Y 2016 Bacterial community dissimilarity between the surface and subsurface soils equals horizontal differences over several kilometers in the western Tibetan Plateau. Environ Microbiol 18:1523–1533. doi:10.1111/1462-2920.13236. 26914676

[31] Zhu GB, Wang SY, Wang Y, Wang CX, Risgaard-Petersen N, Jetten MSM, Yin CQ 2011 Anaerobic ammonia oxidation in a fertilized paddy soil. ISME J 5:1905–1912. doi:10.1038/ismej.2011.63. 21593796 PMC3223303

[32] Wang Y, Zhu GB, Song LY, Wang SY, Yin CQ 2014 Manure fertilization alters the population of ammonia-oxidizing bacteria rather than ammonia-oxidizing archaea in a paddy soil. J Basic Microbiol 54:190–197. doi:10.1002/jobm.201200671. 23686819

[33] Bahram M, Peay KG, Tedersoo L 2015 Local-scale biogeography and spatiotemporal variability in communities of mycorrhizal fungi. New Phytol 205:1454–1463. doi:10.1111/nph.13206. 25767850

[34] Beck S, Powell JR, Drigo B, Cairney JWG, Anderson IC 2015 The role of stochasticity differs in the assembly of soil- and root-associated fungal communities. Soil Biol Biochem 80:18–25. doi:10.1016/j.soilbio.2014.09.010.

[35] Ordynets A, Heilmann-Clausen J, Savchenko A, Bassler C, Volobuev S, Akulov O, Karadelev M, Kotiranta H, Saitta A, Langer E, Abrego N 2018 Do plant-based biogeographical regions shape aphyllophoroid fungal communities in Europe? J Biogeogr 45:1182–1195. doi:10.1111/jbi.13203.

[36] Tsujino M, Hori M, Okuda T, Nakaoka M, Yamamoto T, Noda T 2010 Distance decay of community dynamics in rocky intertidal sessile assemblages evaluated by transition matrix models. Popul Ecol 52:171–180. doi:10.1007/s10144-009-0150-8.

[37] Glassman SI, Wang IJ, Bruns TD 2017 Environmental filtering by pH and soil nutrients drives community assembly in fungi at fine spatial scales. Mol Ecol 26:6960–6973. doi:10.1111/mec.14414. 29113014

[38] Wu WX, Lu HP, Sastri A, Yeh YC, Gong GC, Chou WC, Hsieh CH 2018 Contrasting the relative importance of species sorting and dispersal limitation in shaping marine bacterial versus protist communities. ISME J 12:485–494. doi:10.1038/ismej.2017.183. 29125596 PMC5776463

[39] Shi Y, Li YT, Xiang XJ, Sun RB, Yang T, He D, Zhang KP, Ni YY, Zhu YG, Adams JM, Chu HY 2018 Spatial scale affects the relative role of stochasticity versus determinism in soil bacterial communities in wheat fields across the North China Plain. Microbiome 6:27. doi:10.1186/s40168-018-0409-4. 29402331 PMC5799910

[40] Ramette A, Tiedje JM 2007 Multiscale responses of microbial life to spatial distance and environmental heterogeneity in a patchy ecosystem. Proc Natl Acad Sci U S A 104:2761–2766. doi:10.1073/pnas.0610671104. 17296935 PMC1815255

[41] Hajek M, Rolecek J, Cottenie K, Kintrova K, Horsak M, Poulickova A, Hajkova P, Frankova M, Dite D 2011 Environmental and spatial controls of biotic assemblages in a discrete semi-terrestrial habitat: comparison of organisms with different dispersal abilities sampled in the same plots. J Biogeogr 38:1683–1693. doi:10.1111/j.1365-2699.2011.02503.x.

[42] De Bie T, De Meester L, Brendonck L, Martens K, Goddeeris B, Ercken D, Hampel H, Denys L, Vanhecke L, Van der Gucht K, Van Wichelen J, Vyverman W, Declerck S 2012 Body size and dispersal mode as key traits determining metacommunity structure of aquatic organisms. Ecol Lett 15:740–747. doi:10.1111/j.1461-0248.2012.01794.x. 22583795

[43] Bahram M, Kohout P, Anslan S, Harend H, Abarenkov K, Tedersoo L 2016 Stochastic distribution of small soil eukaryotes resulting from high dispersal and drift in a local environment. ISME J 10:885–896. doi:10.1038/ismej.2015.164. 26394006 PMC4796928

[44] Lee KH, Shaner PJL, Lin YP, Lin SM 2016 Geographic variation in advertisement calls of a Microhylid frog - testing the role of drift and ecology. Ecol Evol 6:3289–3298. doi:10.1002/ece3.2116. 27103987 PMC4833500

[45] Burns AR, Stephens WZ, Stagaman K, Wong S, Rawls JF, Guillemin K, Bohannan B 2016 Contribution of neutral processes to the assembly of gut microbial communities in the zebrafish over host development. ISME J 10:655–664. doi:10.1038/ismej.2015.142. 26296066 PMC4817674

[46] Finlay BJ 2002 Global dispersal of free-living microbial eukaryote species. Science 296:1061–1063. doi:10.1126/science.1070710. 12004115

[47] Soininen J, McDonald R, Hillebrand H 2007 The distance decay of similarity in ecological communities. Ecography 30:3–12. doi:10.1111/j.2006.0906-7590.04817.x.

[48] Vellend M, Srivastava DS, Anderson KM, Brown CD, Jankowski JE, Kleynhans EJ, Kraft NJB, Letaw AD, Macdonald AAM, Maclean JE, Myers-Smith IH, Norris AR, Xue XX 2014 Assessing the relative importance of neutral stochasticity in ecological communities. Oikos 123:1420–1430. doi:10.1111/oik.01493.

[49] Pandit SN, Kolasa J, Cottenie K 2009 Contrasts between habitat generalists and specialists: an empirical extension to the basic metacommunity framework. Ecology 90:2253–2262. doi:10.1890/08-0851.1. 19739387

[50] Li PF, Liu J, Jiang CY, Wu M, Liu M, Li ZP 2019 Distinct successions of common and rare bacteria in soil under humic acid amendment - a microcosm study. Front Microbiol 10:2271. doi:10.3389/fmicb.2019.02271. 31632376 PMC6779779

[51] Umana MN, Zhang CC, Cao M, Lin LX, Swenson NG 2015 Commonness, rarity, and intraspecific variation in traits and performance in tropical tree seedlings. Ecol Lett 18:1329–1337. doi:10.1111/ele.12527. 26415689

[52] Cao LX, Zhang YG, Lu HZ, Yuan JQ, Zhu YY, Liang Y 2015 Grass hedge effects on controlling soil loss from concentrated flow: a case study in the red soil region of China. Soil Till Res 148:97–105. doi:10.1016/j.still.2014.12.009.

[53] Pansu M, Gautheyrou J 2006 Handbook of soil analysis: mineralogical, organic, and inorganic methods. Springer, Berlin, Germany.

[54] Lymperopoulou DS, Adams RI, Lindow SE 2016 Contribution of vegetation to the microbial composition of nearby outdoor air. Appl Environ Microbiol 82:3822–3833. doi:10.1128/AEM.00610-16. 27107117 PMC4907200

[55] Gardes M, Bruns TD 1993 ITS primers with enhanced specificity for basidiomycetes - application to the identification of mycorrhizae and rusts. Mol Ecol 2:113–118. doi:10.1111/j.1365-294x.1993.tb00005.x. 8180733

[56] Caporaso JG, Kuczynski J, Stombaugh J, Bittinger K, Bushman FD, Costello EK, Fierer N, Peña AG, Goodrich JK, Gordon JI, Huttley GA, Kelley ST, Knights D, Koenig JE, Ley RE, Lozupone CA, McDonald D, Muegge BD, Pirrung M, Reeder J, Sevinsky JR, Turnbaugh PJ, Walters WA, Widmann J, Yatsunenko T, Zaneveld J, Knight R 2010 QIIME allows analysis of high-throughput community sequencing data. Nat Methods 7:335–336. doi:10.1038/nmeth.f.303. 20383131 PMC3156573

[57] Magoc T, Salzberg SL 2011 FLASH: fast length adjustment of short reads to improve genome assemblies. Bioinformatics 27:2957–2963. doi:10.1093/bioinformatics/btr507. 21903629 PMC3198573

[58] Bengtsson-Palme J, Ryberg M, Hartmann M, Branco S, Wang Z, Godhe A, De Wit P, Sanchez-Garcia M, Ebersberger I, de Sousa F, Amend AS, Jumpponen A, Unterseher M, Kristiansson E, Abarenkov K, Bertrand YJK, Sanli K, Eriksson KM, Vik U, Veldre V, Nilsson RH 2013 Improved software detection and extraction of ITS1 and ITS2 from ribosomal ITS sequences of fungi and other eukaryotes for analysis of environmental sequencing data. Methods Ecol Evol 4:914–919. doi:10.1111/2041-210X.12073.

[59] Edgar RC, Haas BJ, Clemente JC, Quince C, Knight R 2011 Uchime improves sensitivity and speed of chimera detection. Bioinformatics 27:2194. doi:10.1093/bioinformatics/btr381. 21700674 PMC3150044

[60] Edgar RC 2010 Search and clustering orders of magnitude faster than BLAST. Bioinformatics 26:2460–2461. doi:10.1093/bioinformatics/btq461. 20709691

[61] Weiss S, Xu ZZ, Peddada S, Amir A, Bittinger K, Gonzalez A, Lozupone C, Zaneveld JR, Vazquez-Baeza Y, Birmingham A, Hyde ER, Knight R 2017 Normalization and microbial differential abundance strategies depend upon data characteristics. Microbiome 5:27. doi:10.1186/s40168-017-0237-y. 28253908 PMC5335496

[62] McKnight DT, Huerlimann R, Bower DS, Schwarzkopf L, Alford RA, Zenger KR 2019 Methods for normalizing microbiome data: an ecological perspective. Methods Ecol Evol 10:389–400. doi:10.1111/2041-210X.13115.

[63] Borcard D, Legendre P 2002 All-scale spatial analysis of ecological data by means of principal coordinates of neighbour matrices. Ecol Model 153:51–68. doi:10.1016/S0304-3800(01)00501-4.

[64] Sloan WT, Lunn M, Woodcock S, Head IM, Nee S, Curtis TP 2006 Quantifying the roles of immigration and chance in shaping prokaryote community structure. Environ Microbiol 8:732–740. doi:10.1111/j.1462-2920.2005.00956.x. 16584484

[65] R Development Core Team. 2016 R: a language and environment for statistical computing. R Foundation for Statistical Computing, Vienna, Austria.

[66] Ning DL, Deng Y, Tiedje JM, Zhou JZ 2019 A general framework for quantitatively assessing ecological stochasticity. Proc Natl Acad Sci U S A 116:16892–16898. doi:10.1073/pnas.1904623116. 31391302 PMC6708315

