# Supplementary material for: Spatial Variation in Soil Fungal Communities across Paddy Fields in Subtropical China
Source: mSystems. 2020 Jan 7;5(1):e00704-19. doi: 10.1128/mSystems.00704-19 (PMC6946795; doi:10.1128/mSystems.00704-19)
Supplement: TABLE S1 [file mSystems.00704-19-st001.pdf]

Table S1A. The top panel shows average coefficient of variation (CV) of soil properties in different soil layers, and the bottom panel shows correlations between soil properties and geographical distance in each soil layer. Values of CV are mean ± standardized error. Different letters indicate significant differences at $P < 0.05$. *, **, and *** indicate significant effects at $P < 0.05$, 0.01, and 0.001, respectively.

	Layer 0-10cm	Layer 10-20cm	Layer 20-40cm
Average coefficient of variation			
Average CV (%)	27.05 ± 15.64 a	29.84 ± 16.05 a	38.80 ± 22.72 a
Correlations between soil properties and geographical distance			
SOC	-0.049	0.084	0.190**
TN	-0.001	0.084	0.299***
TP	-0.077	0.001	0.041
C:N	0.104	0.131*	0.216***
TK	-0.006	-0.022	0.026
AN	-0.091	0.075	0.186**
AP	-0.116*	-0.089	0.032
Fe	0.127*	0.231***	0.238***
pH	0.036	0.075	0.143*
CEC	-0.003	0.044	0.029

Table S1B. One-way permANOVA showing the difference of soil properties between soil parent materials.

Variable	*F*	*P*
Soil parent materials	0.733	0.488
