# Supplementary material for: Spatial Variation in Soil Fungal Communities across Paddy Fields in Subtropical China
Source: mSystems. 2020 Jan 7;5(1):e00704-19. doi: 10.1128/mSystems.00704-19 (PMC6946795; doi:10.1128/mSystems.00704-19)
Supplement: TABLE S2 [file mSystems.00704-19-st002.pdf]

Table S2. Correlations between relative abundance of fungal phyla and soil properties. Coefficients were determined by Pearson test. *, **, and *** indicate significant correlations at $P < 0.05$, 0.01, and 0.001, respectively.

	Ascomycota	Zygomycota	Basidiomycota	Chytridiomycota	Glomeromycota	Neocallimastigomycota
SOC	0.393 ***	-0.459 ***	0.096	0.604 ***	0.123	0.167
TN	0.366 ***	-0.427 ***	0.084	0.560 ***	0.144	0.147
TP	0.099	-0.168	0.154	0.229 *	-0.020	0.081
C:N ratio	0.283 *	-0.300 **	0.086	0.270 *	-0.070	0.138
TK	-0.149	0.128	0.001	-0.097	0.098	-0.186
AN	0.376 ***	-0.452 ***	0.143	0.540 ***	0.123	0.135
AP	0.186	-0.336 **	0.387 ***	0.316 **	-0.068	0.190
Fe	-0.426 ***	0.551 ***	-0.415 ***	-0.377 ***	0.111	-0.072
pH	-0.162	0.245 *	-0.109	-0.400 ***	-0.173	-0.222
CEC	0.156	-0.118	-0.179	0.285 *	0.130	0.083
