# Supplementary material for: Spatial Variation in Soil Fungal Communities across Paddy Fields in Subtropical China
Source: mSystems. 2020 Jan 7;5(1):e00704-19. doi: 10.1128/mSystems.00704-19 (PMC6946795; doi:10.1128/mSystems.00704-19)
Supplement: TABLE S3 [file mSystems.00704-19-st003.pdf]

Table S3A. One-way permANOVA showing the difference of fungal communities between soil layers.

	Sørensen's index		Bray-Curtis dissimilarity		Jaccard	
	F	*P*	*F*	*P*	*F*	*P*
Layer 0-10cm **vs** Layer 10-20cm	2.971	< 0.001	2.20	0.004	2.233	< 0.001
Layer 0-10cm **vs** Layer 20-40cm	7.502	< 0.001	6.285	< 0.001	4.984	< 0.001
Layer 10-20cm **vs** Layer 20-40cm	3.527	< 0.001	4.321	< 0.001	2.576	< 0.001

Table S3B. One-way permANOVA showing the effects of soil parent material on fungal community composition.

Variable	Sørensen's index		Bray-Curtis dissimilarity		Jaccard	
	F	*P*	*F*	*P*	*F*	*P*
Soil parent materials	2.239	< 0.001	2.516	< 0.001	1.846	< 0.001
