# Supplementary material for: Spatial Variation in Soil Fungal Communities across Paddy Fields in Subtropical China
Source: mSystems. 2020 Jan 7;5(1):e00704-19. doi: 10.1128/mSystems.00704-19 (PMC6946795; doi:10.1128/mSystems.00704-19)
Supplement: TABLE S4 [file mSystems.00704-19-st004.pdf]

Table S4. Partial Mantel tests showing the correlations between soil properties and beta-diversity indexes of fungal communities. Sørensen: Sørensen's index; Bray-Curtis: Bray-Curtis dissimilarity; Jaccard: Jaccard distance. *, **, and *** indicate significant effects at $P < 0.05$, 0.01, and 0.001, respectively.

	Layer 0-10cm			Layer 10-20cm			Layer 20-40cm		
	Sørensen	Bray-Curtis	Jaccard	Sørensen	Bray-Curtis	Jaccard	Sørensen	Bray-Curtis	Jaccard
SOC	**0.129***	0.032	**0.128***	**0.136***	0.066	**0.193*****	0.036	0.108	0.105
TN	**0.171****	0.011	**0.172****	**0.21*****	**0.115***	**0.211*****	0.035	0.085	**0.128***
TP	**0.191****	**0.151****	**0.189*****	0.077	**0.110***	**0.119***	**0.147****	0.008	**0.211*****
CN	0.029	0.026	0.03	0.076	**0.122***	**0.221*****	0.007	0.045	0.043
TK	0.104	0.149	0.106	**0.120***	0.101	**0.124***	0.069	0.033	0.06
AN	**0.141***	0.054	**0.143***	**0.158****	**0.115***	**0.143****	0.029	0.096	0.044
AP	**0.176****	**0.199*****	**0.179****	**0.220*****	**0.112***	**0.114***	**0.138***	0.045	**0.179****
Fe	0.051	0.01	0.049	**0.115***	**0.127***	0.021	0.029	**0.160****	0.051
pH	**0.235*****	**0.240*****	**0.231*****	**0.277*****	**0.153****	**0.166****	0.108	**0.111***	**0.170****
CEC	0.1	0.019	0.101	0.067	0.041	0.052	0.072	0.02	0.041
