# Supplementary material for: Spatial Variation in Soil Fungal Communities across Paddy Fields in Subtropical China
Source: mSystems. 2020 Jan 7;5(1):e00704-19. doi: 10.1128/mSystems.00704-19 (PMC6946795; doi:10.1128/mSystems.00704-19)
Supplement: TABLE S5 [file mSystems.00704-19-st005.pdf]

Table S5. Random forest model showing the potential important factors influencing OTUs that occurred outside or inside predictions by dominance test. The importance of each predictor was determined by assessing the decrease in prediction accuracy (%IncMSE) when the data for the predictor was randomly permuted. ∑%IncMSE is the accumulated %IncMSE of each significant predictor.

		Environmental factors		Spatial factors		R^2	*P*
		Variable	∑%IncMSE	Variable	∑%IncMSE		
layer 0-10cm	Outside predictions	AP、pH、TP、TN	39.78	PCNM16、PCNM24	27.81	0.179	0.01
	Inside predictions	AP、pH、TP、TK	23.39	PCNM16、PCNM10、PCNM24、PCNM1、PCNM11	30.80	0.187	0.01
layer 10-20cm	Outside predictions	pH、AP、AN、TP、TN、TK、Fe	58.05	PCNM2、PCNM16、PCNM25、PCNM11、PCNM24、PCNM9、PCNM1、PCNM10、PCNM7、PCNM13、PCNM8	83.89	0.365	0.01
	Inside predictions	TK、AP	8.64	PCNM2、PCNM16、PCNM5、PCNM20、PCNM18、PCNM25、PCNM14、PCNM23、PCNM24	69.90	0.232	0.01
layer 20-40cm	Outside predictions	pH、CEC、SOC、TN、TK	28.96	PCNM22、PCNM16、PCNM18、PCNM24	19.79	0.146	0.01
	Inside predictions	Fe、SOC、TN、pH、CN、CEC	17.66	PCNM22、PCNM17、PCNM18、PCNM25、PCNM1、PCNM7	19.87	0.125	0.01
