# Supplementary figures and images for: Spatial Variation in Soil Fungal Communities across Paddy Fields in Subtropical China
Source: mSystems. 2020 Jan 7;5(1):e00704-19. doi: 10.1128/mSystems.00704-19 (PMC6946795; doi:10.1128/mSystems.00704-19)

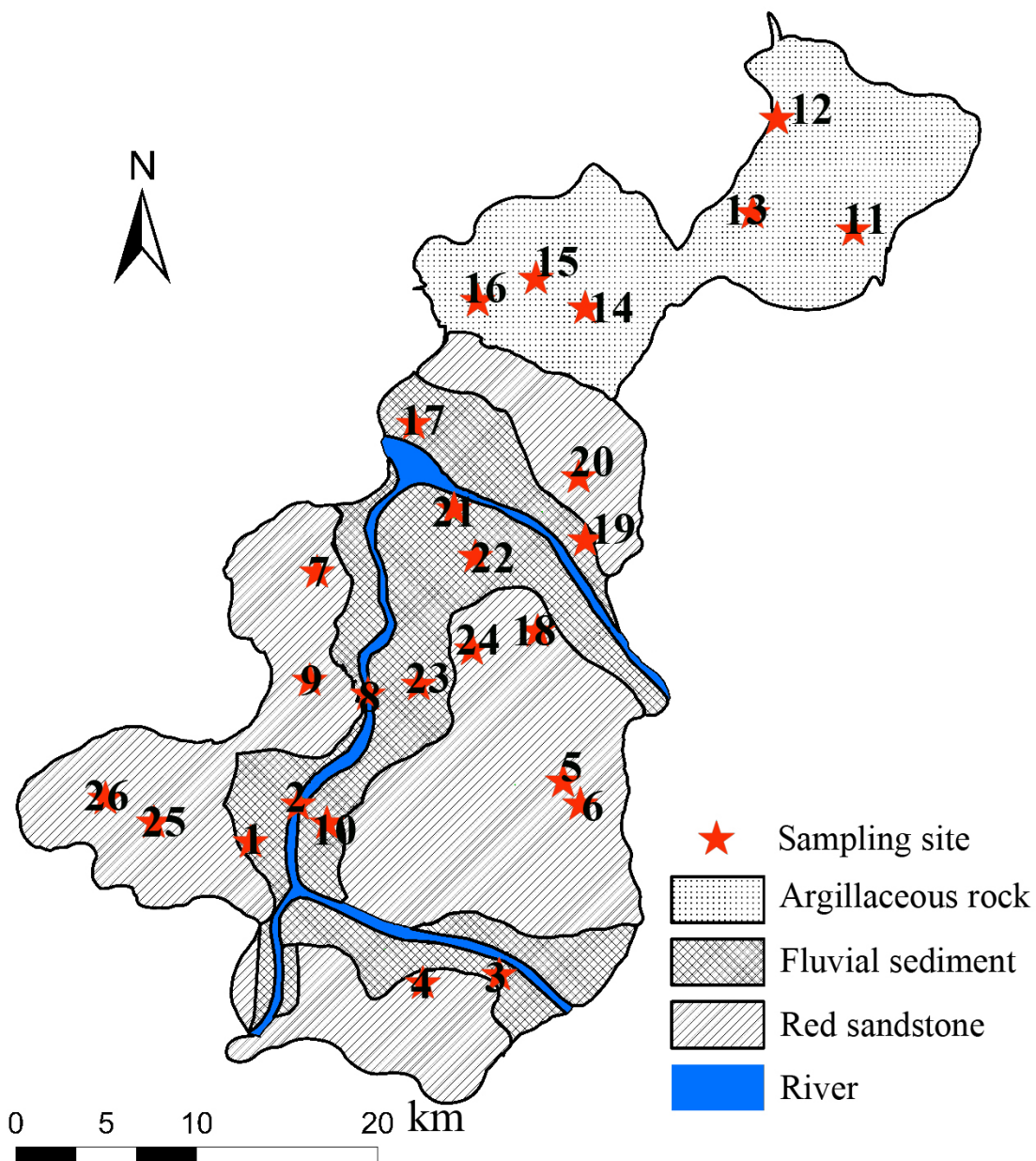

| Site | longitude | latitude   |
|------|-----------|------------|
| 1    | 28.2097 N | 116.8010 E |
| 2    | 28.2289 N | 116.8260 E |
| 3    | 28.1428 N | 116.9230 E |
| 4    | 28.1397 N | 116.8850 E |
| 5    | 28.2385 N | 116.9560 E |
| 6    | 28.2290 N | 116.9640 E |
| 7    | 28.3465 N | 116.8330 E |
| 8    | 28.2832 N | 116.8580 E |
| 9    | 28.2875 N | 116.8390 E |
| 10   | 28.2194 N | 116.8380 E |
| 11   | 28.5136 N | 117.1020 E |
| 12   | 28.5695 N | 117.0620 E |
| 13   | 28.5231 N | 117.0510 E |
| 14   | 28.4714 N | 116.9690 E |
| 15   | 28.4809 N | 116.9450 E |
| 16   | 28.4773 N | 116.9130 E |
| 17   | 28.4156 N | 116.8810 E |
| 18   | 28.3141 N | 116.9430 E |
| 19   | 28.3612 N | 116.9700 E |
| 20   | 28.3868 N | 116.9670 E |
| 21   | 28.3727 N | 116.9020 E |
| 22   | 28.3505 N | 116.9120 E |
| 23   | 28.2885 N | 116.8830 E |
| 24   | 28.3045 N | 116.9110 E |
| 25   | 28.2189 N | 116.7510 E |
| 26   | 28.2289 N | 116.7320 E |

Supplement: FIG S1 [file mSystems.00704-19-sf001.pdf]

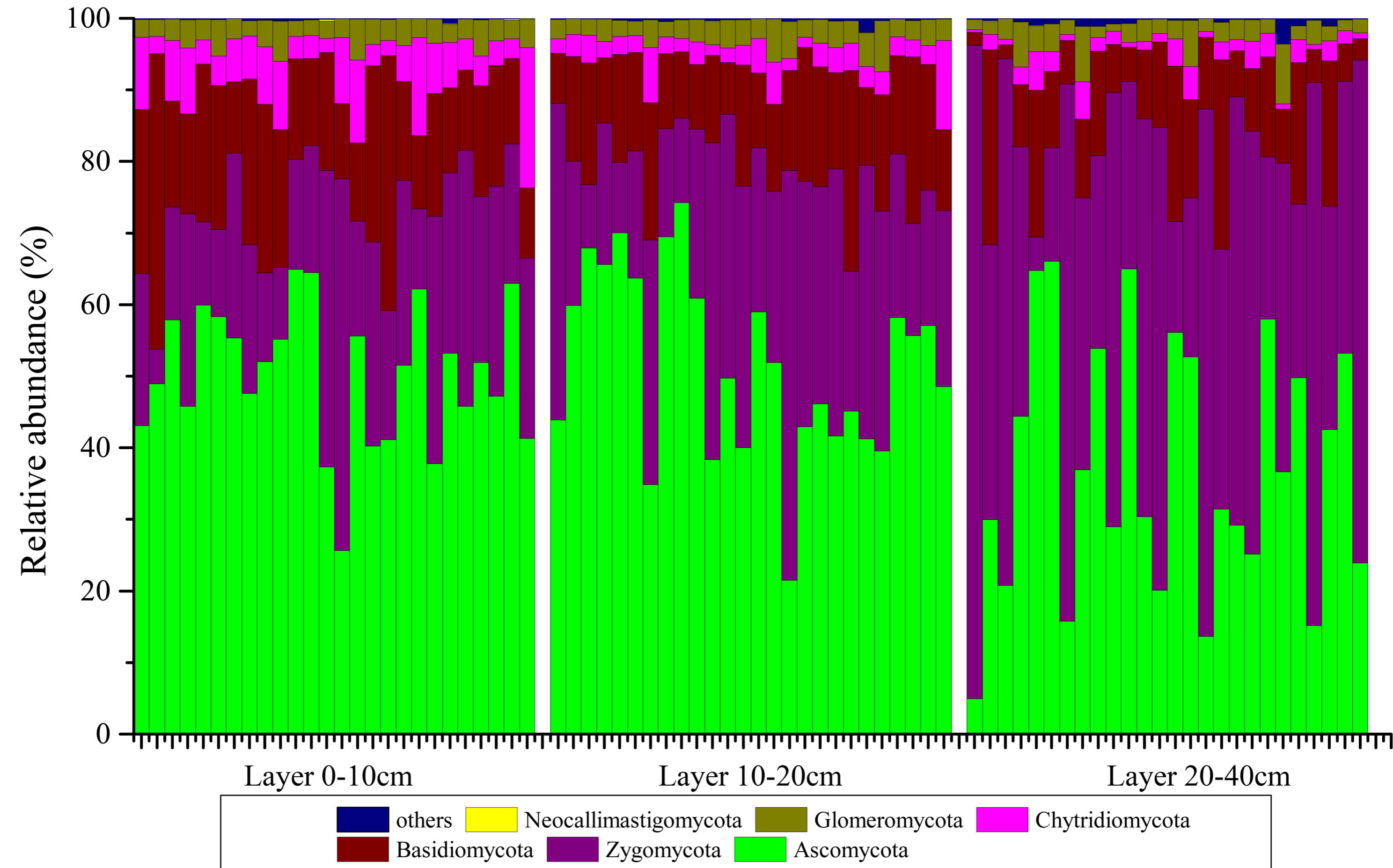

Supplement: FIG S2 [file mSystems.00704-19-sf002.pdf]

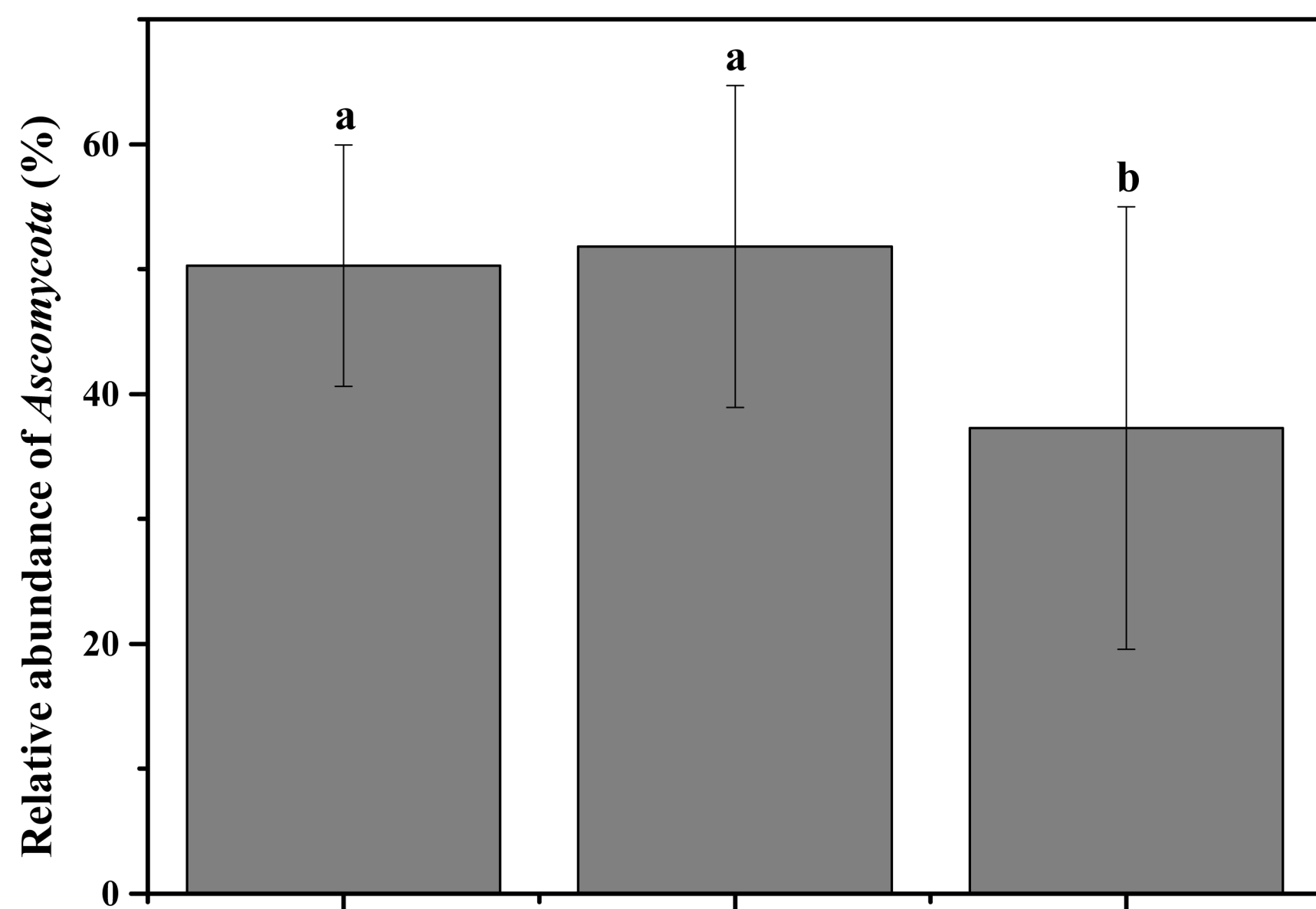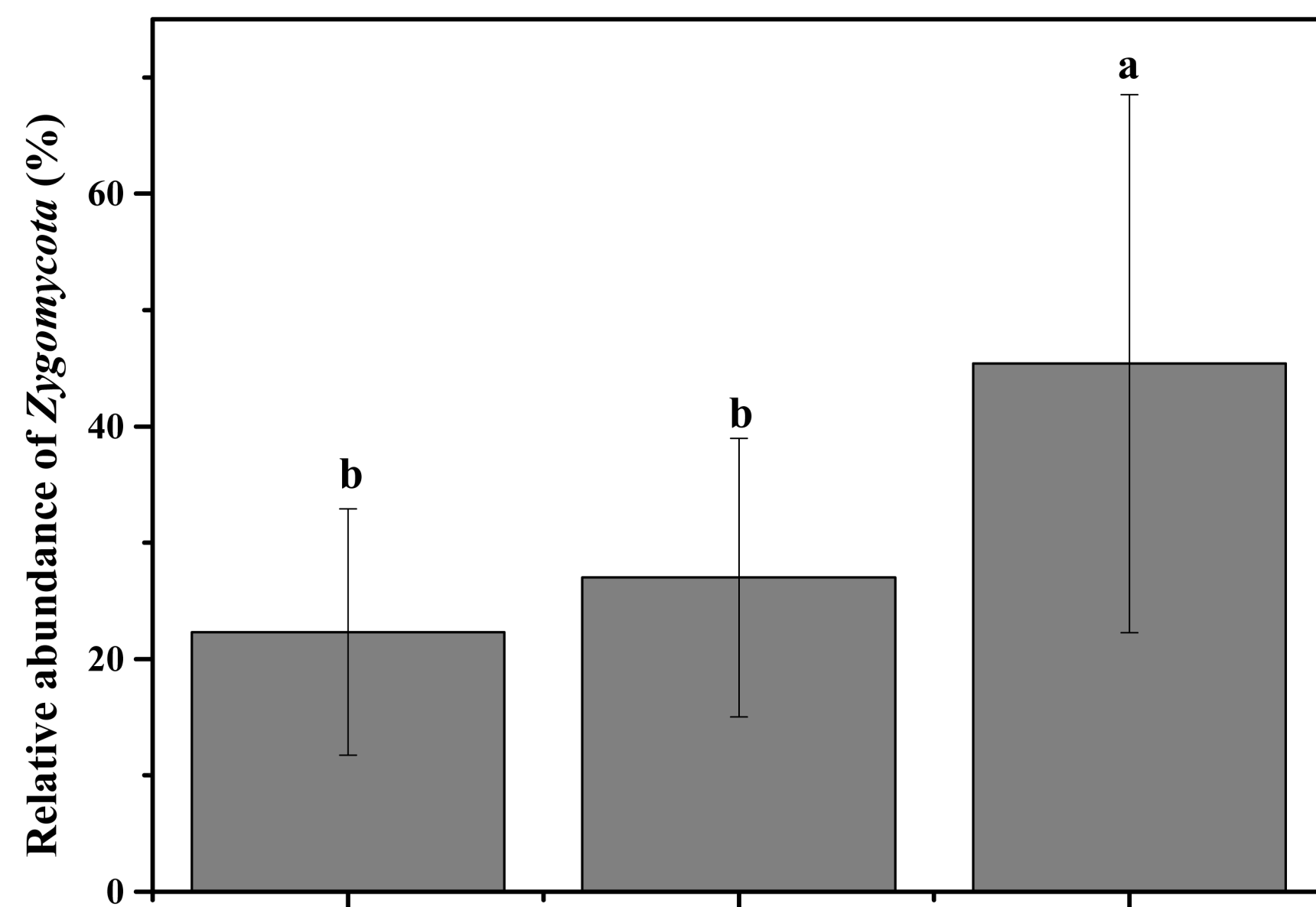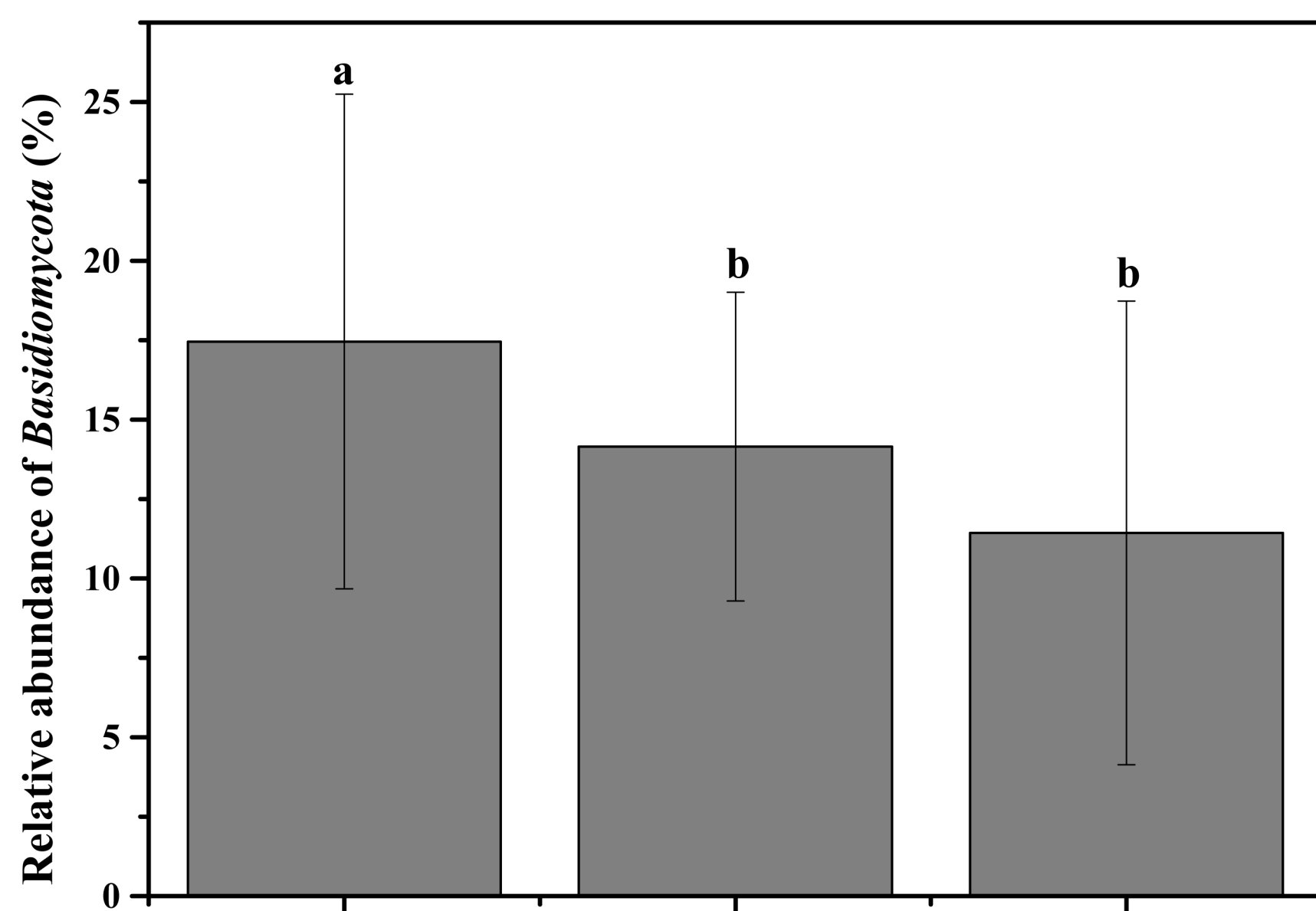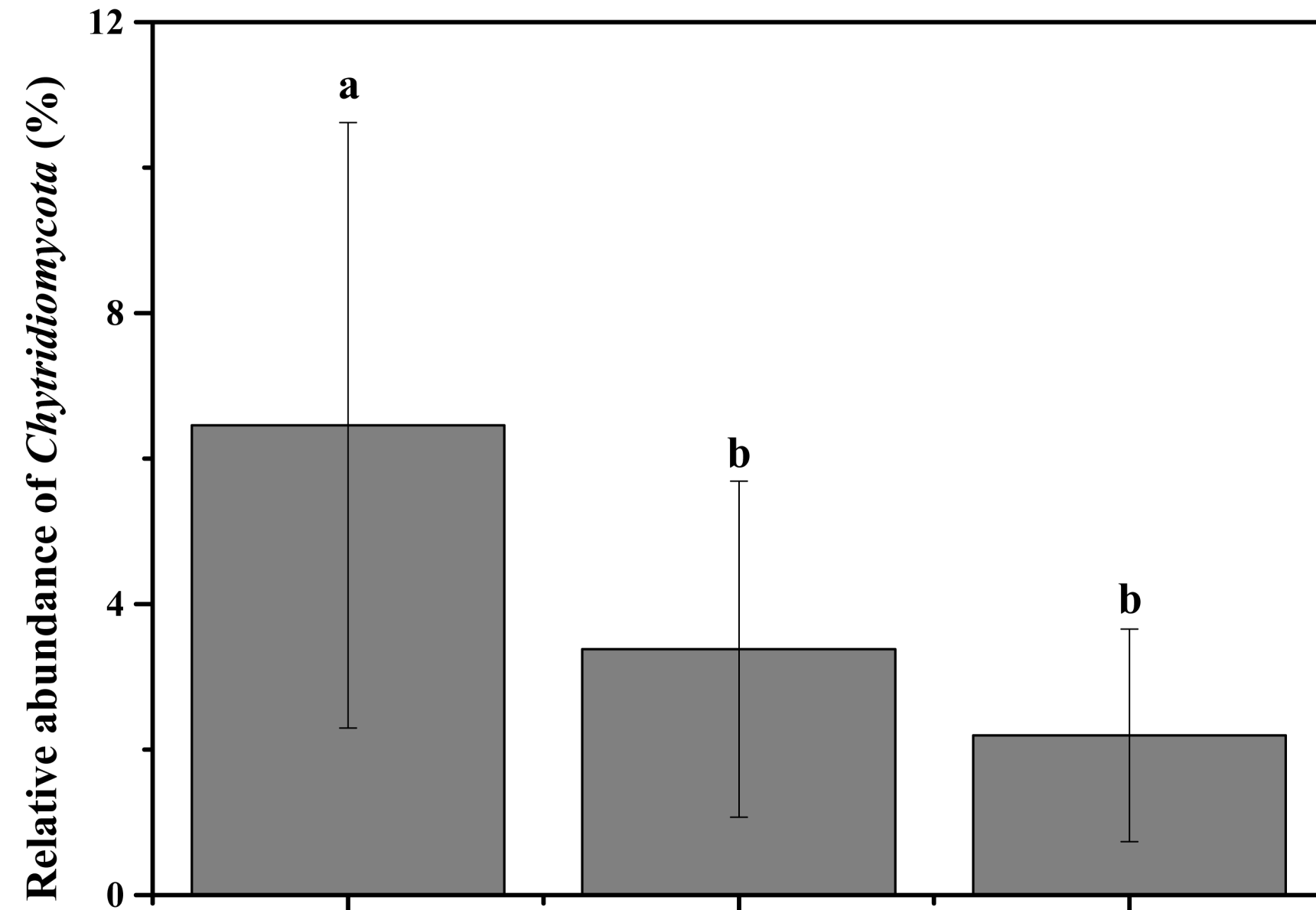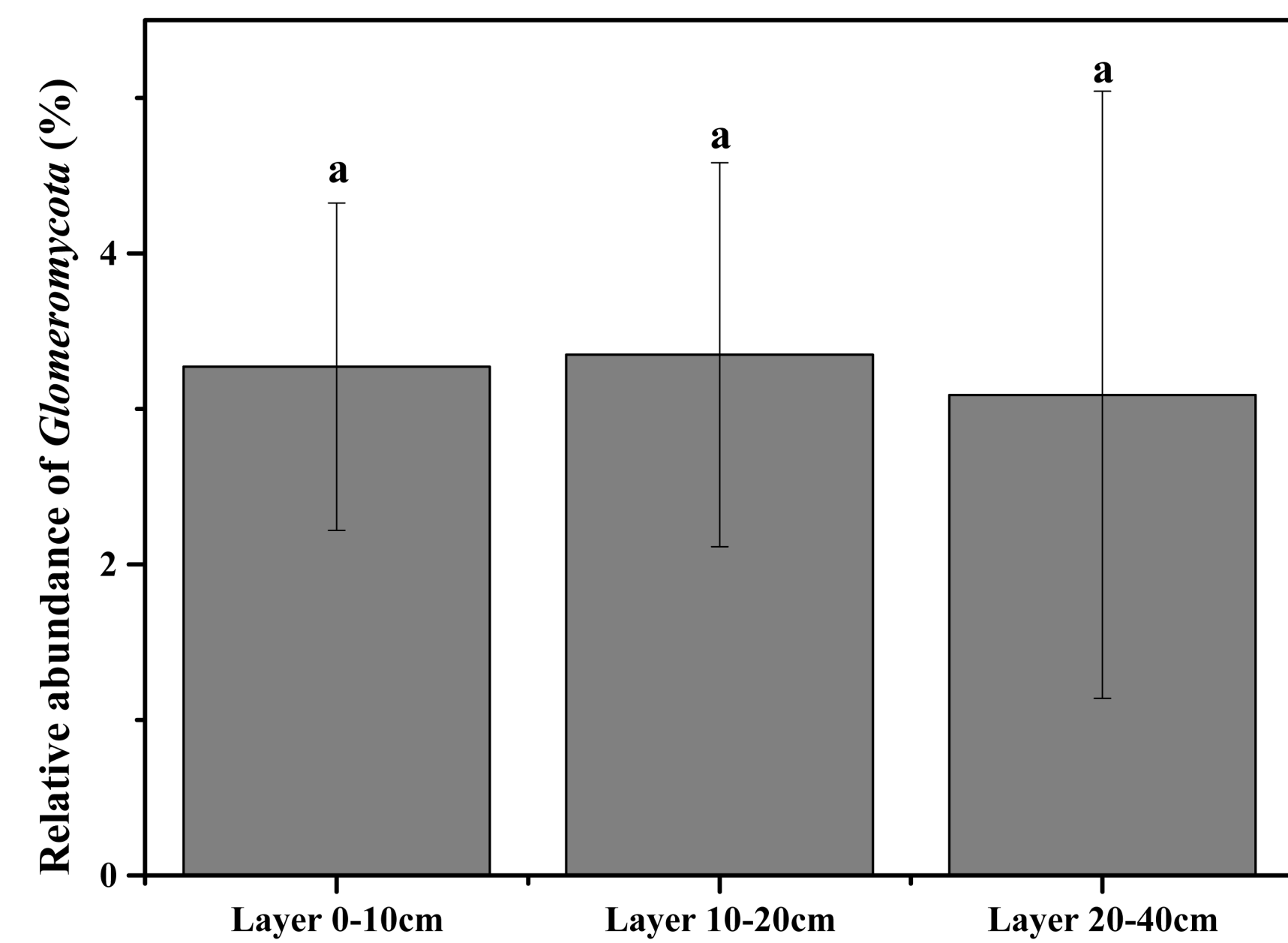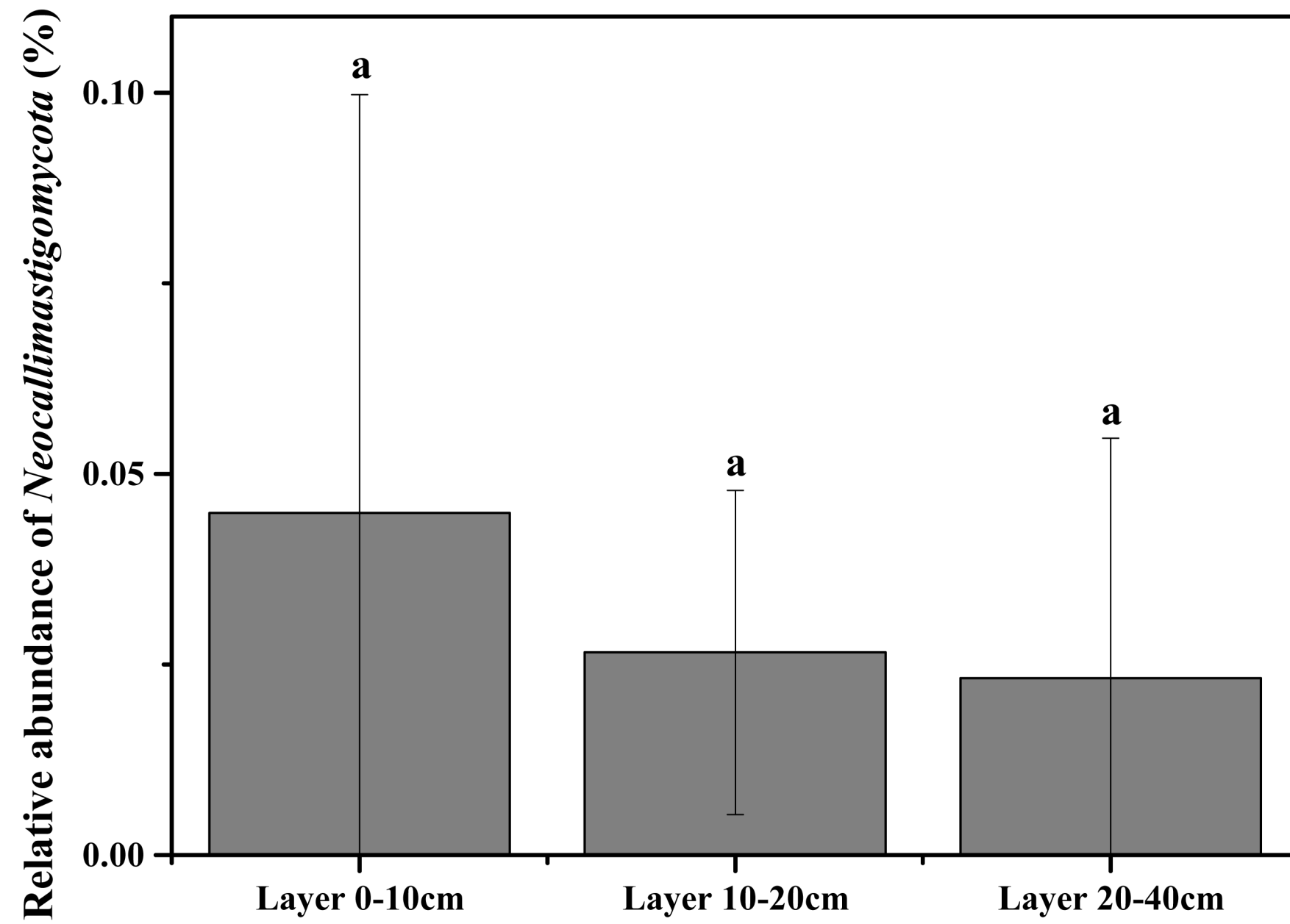

Supplement: FIG S3 [file mSystems.00704-19-sf003.pdf]

# Sorensen's index

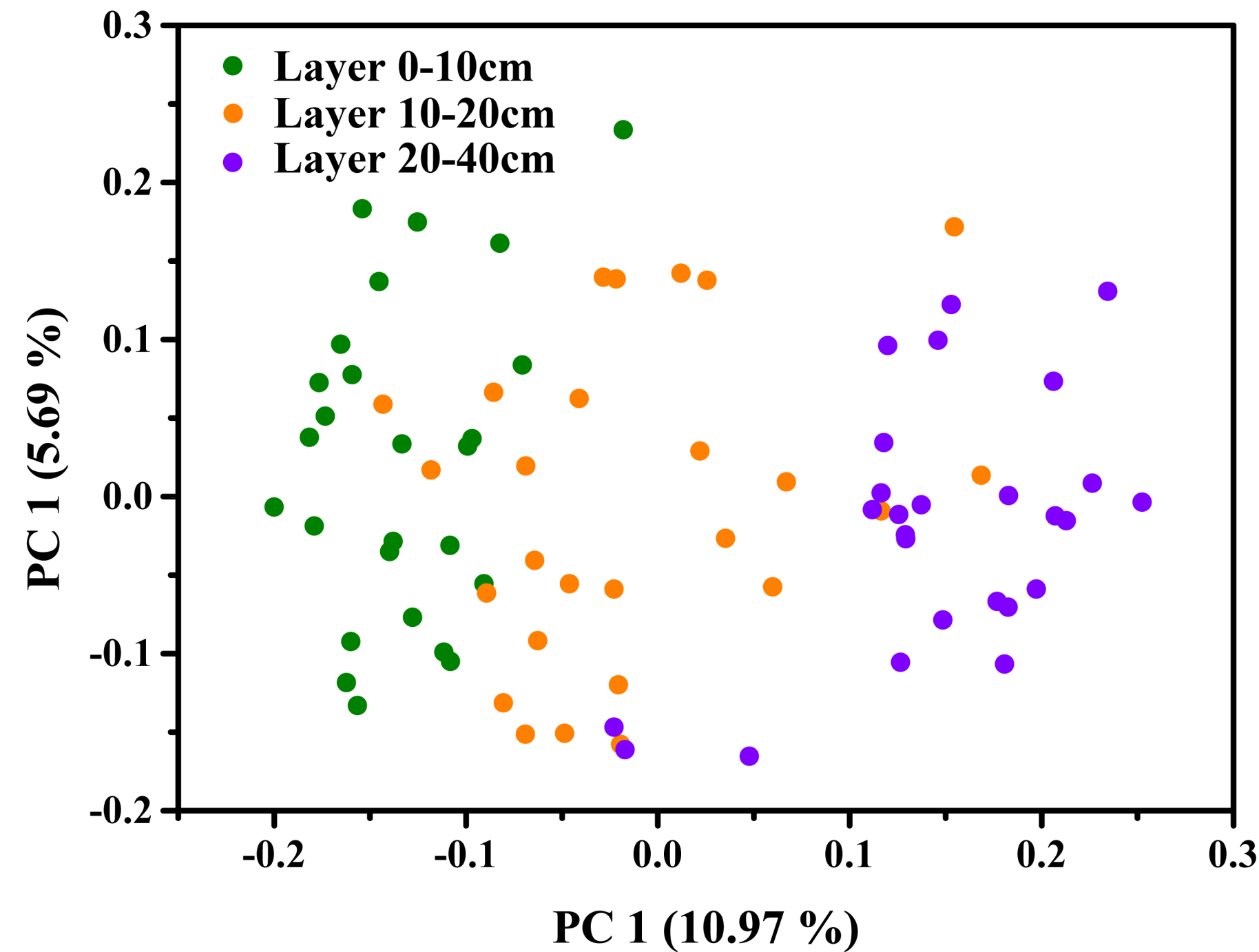

# Bray-Curtis dissimilarity

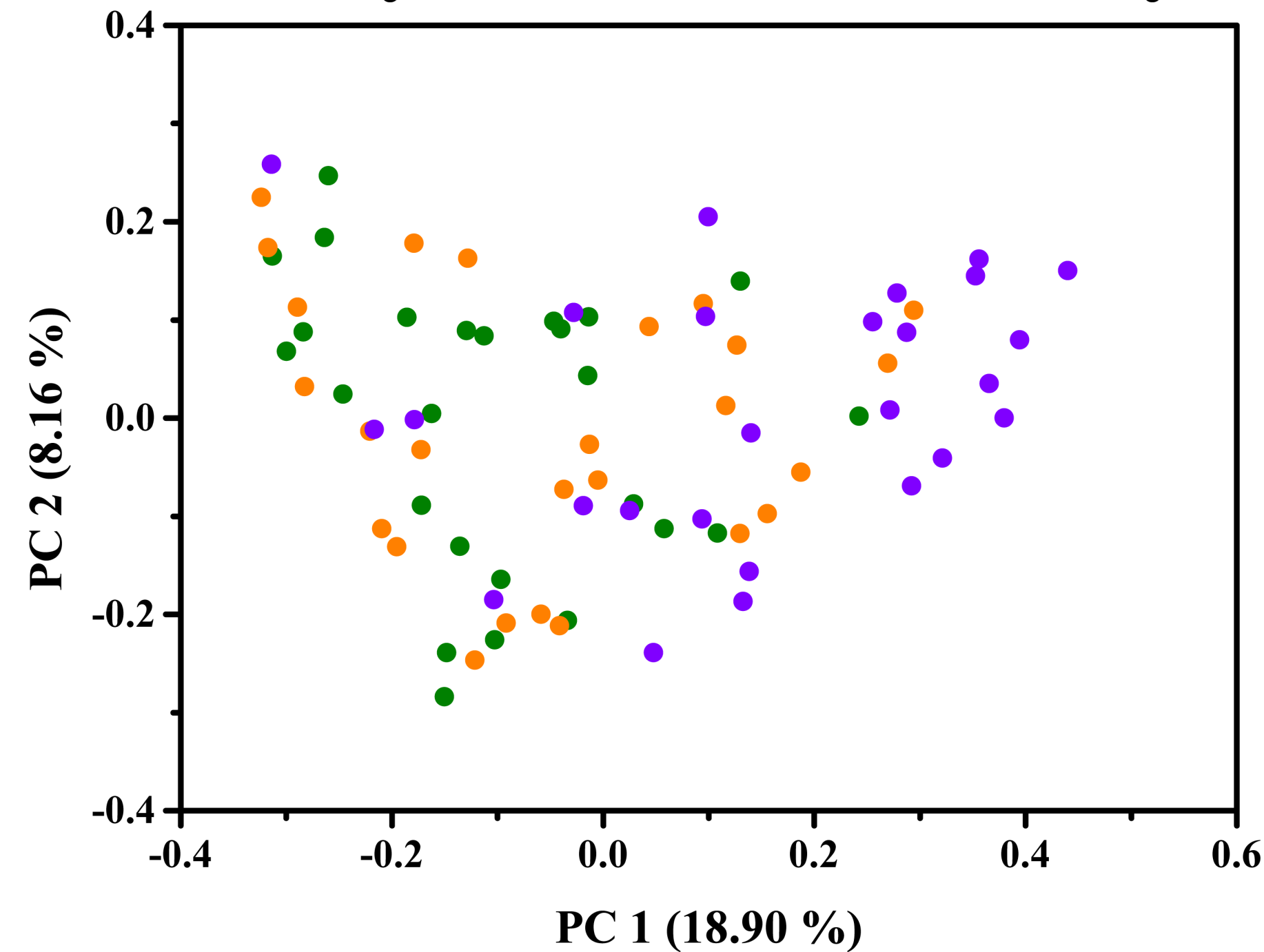

# Jaccard distance

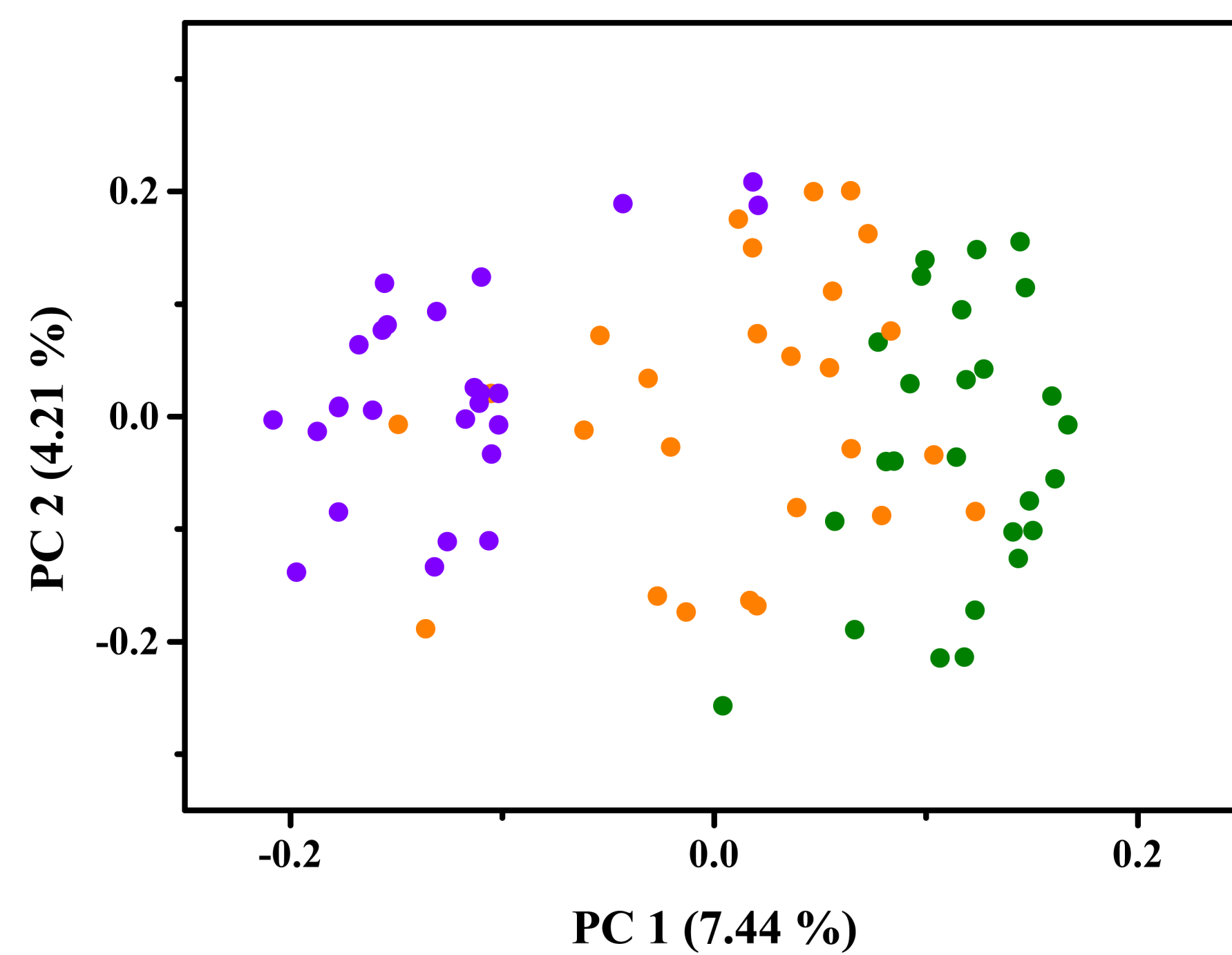

Supplement: FIG S4 [file mSystems.00704-19-sf004.pdf]

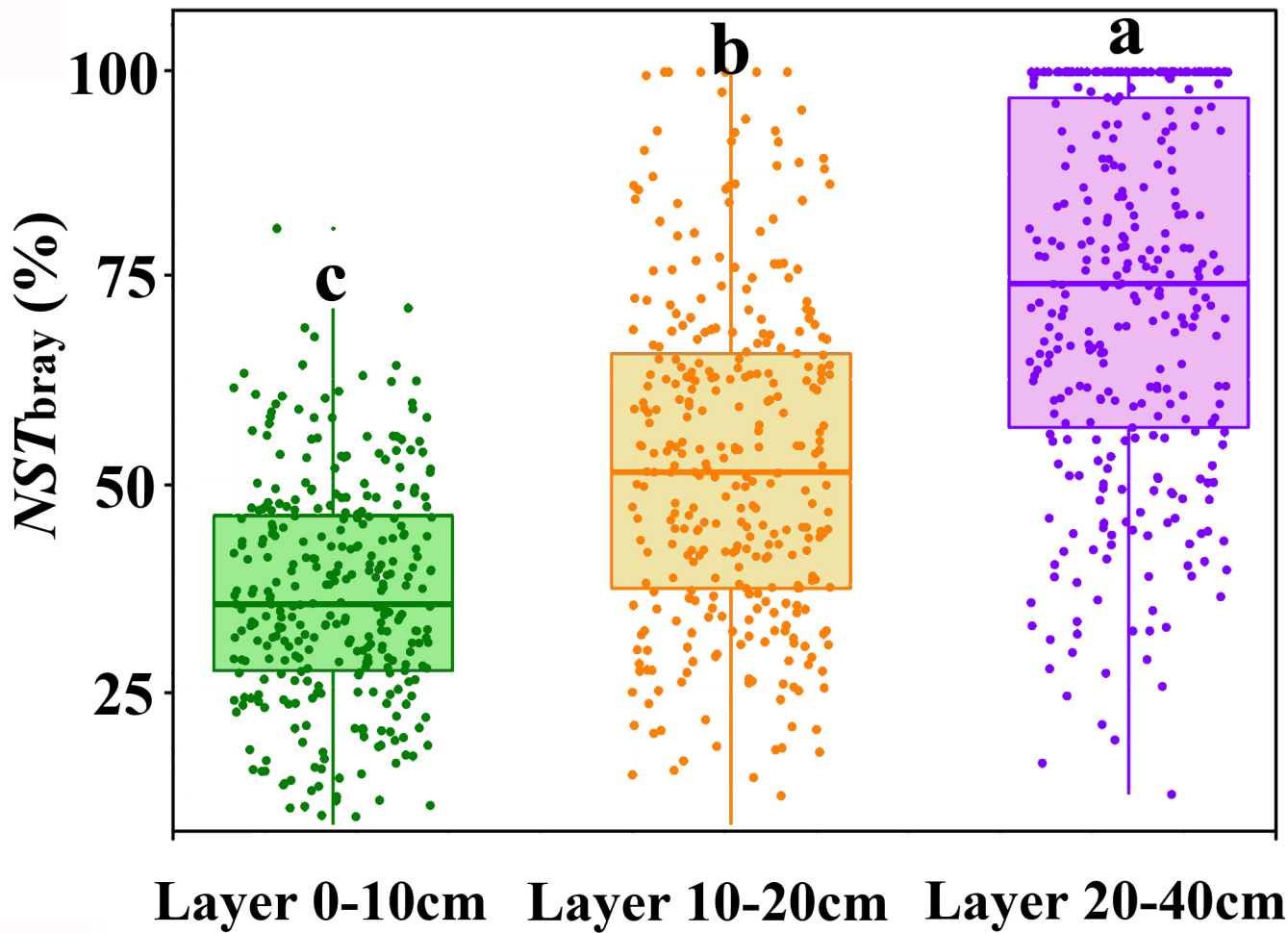

Supplement: FIG S5 [file mSystems.00704-19-sf005.pdf]
